# People’s political views, perceived social norms, and individualism shape their privacy concerns for and acceptance of pandemic control measures that use individual-level georeferenced data

**DOI:** 10.1186/s12942-023-00354-3

**Published:** 2023-12-06

**Authors:** Mei-Po Kwan, Jianwei Huang, Zihan Kan

**Affiliations:** 1 Department of Geography and Resource Management, Institute of Space and Earth Information Science, and Institute of Future Cities, The Chinese University of Hong Kong, Shatin, Hong Kong, China; 2 Institute of Space and Earth Information Science, The Chinese University of Hong Kong, Shatin, Hong Kong, China

**Keywords:** Individual-level georeferenced data, Privacy concerns, Individualism, Social norms, Political views

## Abstract

**Background:**

As the COVID-19 pandemic became a major global health crisis, many COVID-19 control measures that use individual-level georeferenced data (e.g., the locations of people’s residences and activities) have been used in different countries around the world. Because these measures involve some disclosure risk and have the potential for privacy violations, people’s concerns for geoprivacy (locational privacy) have recently heightened as a result, leading to an urgent need to understand and address the geoprivacy issues associated with COVID-19 control measures that use data on people’s private locations.

**Methods:**

We conducted an international cross-sectional survey in six study areas (n = 4260) to examine how people’s political views, perceived social norms, and individualism shape their privacy concerns, perceived social benefits, and acceptance of ten COVID-19 control measures that use individual-level georeferenced data. Multilevel linear regression models were used to examine these effects. We also applied multilevel structure equation models (SEMs) to explore the direct, indirect, and mediating effects among the variables.

**Results:**

We observed a tradeoff relationship between people’s privacy concerns and the acceptance (and perceived social benefits) of the control measures. People’s perceived social tightness and vertical individualism are positively associated with their acceptance and perceived social benefits of the control measures, while horizontal individualism has a negative association. Further, people with conservative political views and high levels of individualism (both vertical and horizontal) have high levels of privacy concerns.

**Conclusions:**

Our results first suggest that people’s privacy concerns significantly affect their perceived social benefits and acceptance of the COVID-19 control measures. Besides, our results also imply that strengthening social norms may increase people’s acceptance and perceived social benefits of the control measures but may not reduce people’s privacy concerns, which could be an obstacle to the implementation of similar control measures during future pandemics. Lastly, people’s privacy concerns tend to increase with their conservatism and individualism.

**Supplementary Information:**

The online version contains supplementary material available at 10.1186/s12942-023-00354-3.

## Background

In the past decade or so, the rapid advances and widespread use of a wide range of geospatial technologies (e.g., GPS-equipped mobile phones and sensors) in people’s daily life have ushered in the era of geospatial big data. With the help of these location-aware technologies, enormous amounts of data containing information on accurate geographic locations (georeferenced data) can be collected and used for understanding many geographic, social, and health-related phenomena (e.g., disease outbreaks) [1, 2]. Nevertheless, people’s identities could be disclosed based on the georeferenced data through *spatial reverse engineering*, disclosing specific individuals’ identities by linking the georeferenced data with other data sources (e.g., census or survey data) [3–7]. The potential benefits of using georeferenced data for addressing urgent social and health issues may thus be undermined by people’s concerns for their locational privacy (geoprivacy).

As the COVID-19 pandemic became a major global health crisis [8], many countries have implemented control measures that use individual-level georeferenced data (IGD) (e.g., the locations of infected persons’ residences and activities). There are three main types of COVID-19 control measures that use IGD, which may be used to discover people’s identity: (1) contact tracing methods that identify the persons who had been in close contact with infected persons or visited the places or venues infected persons recently visited; (2) methods that monitor whether people required to self-quarantine are adhering to the requirements (i.e., staying where they are supposed to be); and (3) methods that disclose (and often publicize) the places or venues visited by infected persons so that other people may avoid visiting these locations and venues [9–13]. Many countries have implemented some variants of these location-based COVID-19 control measures. However, these measures pose a certain threat to people’s geoprivacy because the identity of an infected person may be accurately recovered through spatial reverse engineering by linking the IGD to publicly available data. This is particularly possible when pertinent demographic information (e.g., age, gender, or occupation) of infected persons is released along with the IGD.

Further, COVID-19 control methods that collect and use IGD may pose a serious geoprivacy threat because recent advances in geospatial artificial intelligence (GeoAI) and high-performance computing may significantly increase the accuracy of spatial reverse engineering [14, 15]. As a result, the effectiveness of COVID-19 control measures that use IGD may be significantly influenced by people’s acceptance of these measures, which in turn may be affected by people’s perceptions of the potential of these measures for violating their geoprivacy and the social benefits of these measures [16]. In this light, people’s privacy concerns about COVID-19 control measures may be an important determinant of the effectiveness of these measures. There is thus an urgent need to understand and address the geoprivacy issues associated with COVID-19 control measures that use the georeferenced data of people’s residences and activities. Further, although the World Health Organization has declared that COVID-19 is no longer a global health emergency, understanding people’s privacy concerns for COVID-19 control measures is still critical because similar measures may be used to control future pandemics.

However, no large-scale study to date has addressed the geoprivacy issues related to COVID-19 control measures and the effects of people’s privacy concerns on the acceptability and potential effectiveness of these measures. We thus have very limited knowledge about how people’s privacy concerns are related to what COVID-19 control measures are more likely to be accepted, effective, or successful. Further, behavioral scientists have found that people’s willingness to adhere to COVID-19 control measures (e.g., wearing masks and social distancing) was significantly affected by social and cultural factors. For instance, Bavel et al. [17] suggested that people’s behavior is influenced by social norms and some differences in people’s responses to the COVID-19 pandemic may be understood in terms of cultural differences. As Bavel et al. [17] argued, compared to people in Asian countries, people in Western European and North American countries are less willing to adhere to COVID-19 control measures since these measures require them to relinquish some individual freedom (individualism). On the other hand, Asian cultures tend to value individuals’ obligations to the larger society (collectivism) more than Western European and North American cultures, and this may lead to a lower interpersonal transmission risk of COVID-19. Habersaat et al. [18] also proposed similar ideas that it was necessary to consider balancing individual rights with the social good in the implementation of control measures during the pandemic. Specifically, they argued that people in collectivist countries are more willing to act for the larger society, while people in individualistic countries tend to prioritize individual benefits.

This study is the first major international study that examines people’s geoprivacy concerns for and acceptance of COVID-19 control measures that use individual-level georeferenced data (IGD). Using online surveys, we collected data from 4,260 participants in six study areas (the U.S., the U.K., New Zealand, Hong Kong, Japan, and South Korea) to answer the following questions: (1) What COVID-19 control measures are more likely to be effective or successful based on the levels of people’s acceptance of these measures? (2) Are people’s privacy concerns, perceived social benefits, and acceptance of COVID-19 control measures related to their individualist orientation, political views, and the strengths of social norms in the study areas? The findings will enhance our understanding of the influence of people’s political views and cultural and social factors (e.g., individualistic orientation and the strengths of and adherence to social norms) on the overall effectiveness of COVID-19 control measures in different countries. They can also help us understand how people’s geoprivacy concerns may be related to the acceptability and potential effectiveness of other government measures or policies that use IGD in other situations or contexts (e.g., future pandemics).

## Methods

**Fig. 1:**
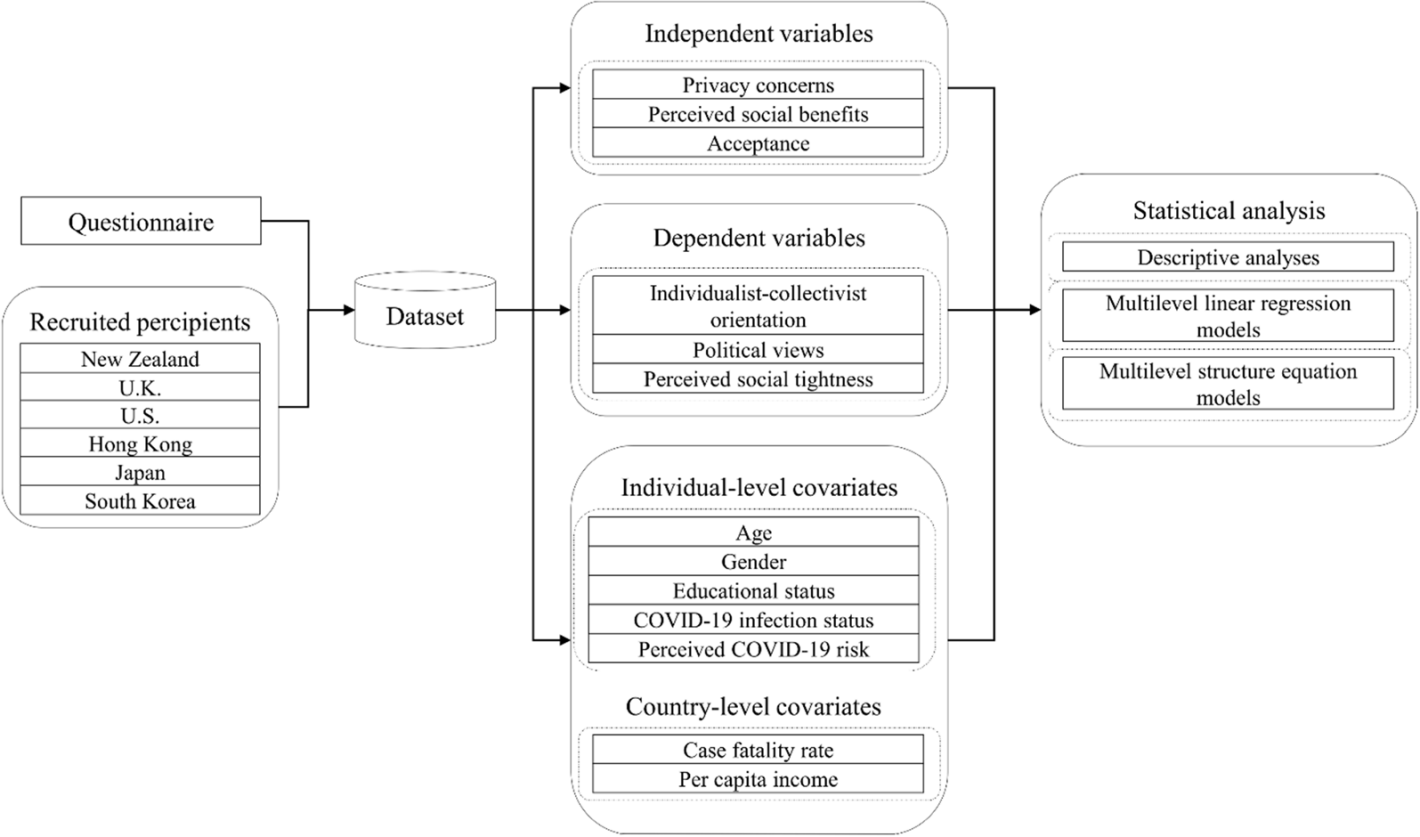
Research flow

In this work, we designed a research flow (see Fig. 1) to address the abovementioned research questions. First, we constructed a questionnaire with various items to measure people’s privacy concerns, perceived social benefits, and acceptance of COVID-19 control measures, and their individualist orientation, political views, perceived social tightness, and perceived COVID-19 risk according to previous studies. Then, we conducted an online survey to recruit 4,260 participants from six study areas to collect the dataset. After that, we applied descriptive analyses to address the first research question. Finally, we used multilevel linear regression models and multilevel structure equation models (SEMs) to address the second research question.

### Study areas and data collection

We used online surveys to collect data from September 8 to 16, 2022, from a target sample of 4,200 participants in six study areas: New Zealand, the U.K., the U.S., Hong Kong, Japan, and South Korea. These study areas have implemented some COVID-19 control measures that use IGD (e.g., South Korea has used the mobile phone locations of infected persons when conducting contact tracing, and Hong Kong has utilized electronic wristbands to ensure those required to self-quarantine adhere to the requirement). There are considerable cultural differences between the first three study areas (Western) and the latter three study areas (Eastern) (e.g., North American cultures tend to be individualistic while Asian societies tend to prioritize commitment to social norms; Bavel et al. [17]). They also have different experiences with the COVID-19 pandemic and levels of success in controlling it (e.g., New Zealand is considered highly successful, while the U.S. is considered far from satisfactory). It should be noted that the six study areas had experienced large-scale outbreaks of omicron at the time of the survey (i.e., September 8 to 16, 2022), and the control measures had shifted from non-pharmaceutical interventions to vaccination. These six study areas thus entail some diversity in the control measures implemented, cultural contexts, and experiences of the pandemic.

For each of the six study areas, data were collected through an online survey from 700 individuals who are at least 18 years old and have lived in the study area during the COVID-19 pandemic (i.e., the total target sample is 4,200 individuals). The surveys were implemented by an international survey research company (Cint) using quota sampling to ensure the representativeness of the samples. Solicitations to participate in the study were distributed via Cint’s international networks in each of the six study areas. As indicated by the Winton Centre for Risk and Evidence Communication (WCREC 2020) [19], which has completed an international online survey on people’s attitudes towards the risk of COVID-19, 700 participants per study area would achieve a margin of error of + /‒ 4% and yield reasonably reliable results. A total of 4,260 valid responses were finally obtained. The Survey and Behavioral Research Ethics (SBRE) Committee of the authors’ universities reviewed and approved the study protocol and survey questionnaire. Table 1 shows the sociodemographic profile of the participants in the six study areas. Specifically, the distribution of gender represents their respective populations well. In terms of age group, our sample has a higher percentage of young adults (25–45 years old) for the six study areas.

**Table 1:** Sociodemographic characteristics of survey participants, and comparison with those of the national/urban populations (n = 4260)

		**US**		**UK**		**NZ**	
		Sample (n%)	Population (n%) ^a^	Sample (n%)	Population (n%) ^b^	Sample (n%)	Population (n%) ^c^
Age	18–24	11	18	9	14	11	16
	25–45	41	33	46	32	43	34
	45 +	48	49	45	54	46	50
Gender	Male	48	49	47	49	49	49
	Female	52	51	53	51	51	51

		HK		JP		SK	
		Sample (n%)	Population (n%) ^d^	Sample (n%)	Population (n%) ^e^	Sample (n%)	Population (n%) ^f^
Age	18–24	6	9	5	11	7	12
	25–45	54	33	33	26	47	31
	45 +	40	58	62	63	45	57
Gender	Male	47	44	49	49	50	50
	Female	53	56	51	51	50	50

^a^2021 the United States Census Bureau data (15 + years old)

^b^2021 the United Kingdom Office for National Statistics data (15 + years old)

^c^2021 New Zealand Statistics Bureau data

^d^2021 Hong Kong Statistics Bureau data (15 + years old)

^e^2020 Japan statistics bureau data (15 + years old)

^f^2022 South Korea statistics bureau data (15 + years old)

### Variables and factor analysis

Six versions of the survey questionnaire were prepared with the help of the collaborators of the project who are native (or close-to-native) academics and have considerable exposure to the social and cultural contexts in the respective study areas. All versions of the questionnaire asked the same questions. They only have minor variations to ensure that the expressions are socially and culturally appropriate to the study area in question (e.g., the name of the local currency). The survey questionnaire has three sections.

#### Privacy concerns, perceived social benefits, and acceptance

We collected participants’ data on their views of ten COVID-19 control measures that rely on individual-level georeferenced data (IGD) using the questionnaire. The ten measures can be classified into three broad types: contact tracing, self-quarantine monitoring, and location disclosure (i.e., disclosure of visited places, locations, or venues to the public) (see Additional file 1: Table S1 for details). For each of these ten COVID-19 control measures, the questionnaire collects data from participants concerning: (1) their privacy concerns for the measure, (2) their perceived social benefits of the measure (i.e., the level of social benefits the respondent thinks would be gained by providing the information requested by public health agencies) and (3) their acceptance of the measure. Each of these three response items is measured on a 7-point scale (from 1 to 7). For privacy concerns, “1” indicates “not concerned at all,” 4 indicates “neutral,” and 7 indicates “very concerned.” For perceived social benefits, “1” indicates “not beneficial at all,” while “7” indicates “very beneficial.” For acceptance, “1” indicates “not acceptable at all,” while “7” indicates “very acceptable.” The Cronbach’s alphas of the response items for privacy concerns, perceived social benefits, and acceptance are 0.918, 0.940, and 0.928 respectively, indicating that they have good internal consistency. We further derive a total score for each category of response items (by summing all scores of the response items for privacy concerns, perceived social benefits, and acceptance respectively). A higher total score indicates a higher level of privacy concerns, perceived social benefits, or acceptance for a participant. Additional file 1: Figure S1 shows the statistical distribution of people’s privacy concerns, perceived social benefits, and acceptance of COVID-19 control measures in the six study areas.

#### Individualist-collectivist orientation

We used 16 items to assess participants’ individualist-collectivist orientation (see Table 2). These 16 items were designed by Triandis and Gelfand [20] and can be used to assess participants’ personal individualist and collectivist orientations. In other words, these 16 items are not used to assess the participants’ perceived individualist and collectivist orientation of the area in which they live (thus, unlike House et al. [21]). Specifically, participants assessed their agreement on the 16 items using a 7-point scale (from 1 to 7), where a higher number stands for a higher level of agreement. Further, these 16 items cover four distinct patterns of individualist and collectivist orientation: vertical individualism, horizontal individualism, vertical collectivism, and horizontal collectivism. A vertical-individualist person tends to be concerned with improving his/her individual status among others via competition and achievement, whereas a horizontal-individualist person tends to view himself or herself as equal to others in status but emphasizes her/her uniqueness and distinctiveness from others in the group. A vertical-collectivist person prioritizes his/her in-group goals over personal goals, while a horizontal-collectivist person values sociability and interdependence within an egalitarian framework. We used exploratory factor analysis (EFA) to evaluate and validate the four-factor structure underlying the 16 items. We expected that people’s vertical and horizontal individualist-collectivist scores would be explained by four underlying factors at the individual level with item loadings in the expected direction. Table 2 presents the items and factor loadings. As expected, the EFA results demonstrated a four-factor solution: one factor with 3 items assessing vertical individualism (eigenvalue = 4.52), one factor with 3 items assessing horizontal individualism (eigenvalue = 1.96), one factor with 4 items assessing vertical collectivism (eigenvalue = 1.57), and one factor with 3 items assessing horizontal collectivism (eigenvalue = 1.13). Notably, a person’s individualist orientation and collectivist orientation are mirror concepts. In other words, the individualist orientation score is strongly negatively associated with the collectivist orientation score. We therefore applied participants’ vertical and horizontal individualism in the study. Higher vertical or horizontal individualism scores mean that the participant has a stronger vertical or horizontal individualist orientation. Additional file 1: Figures S2(a) and S2(b) present the distribution of participants’ vertical and horizontal individualism in the six study areas. They indicate that participants from Western study areas (i.e., the U.S., the U.K., and New Zealand) have lower vertical individualism scores and higher horizontal individualism scores than those from Eastern study areas (i.e., Hong Kong, Japan, and South Korea).

**Table 2:** Questions and factor loadings for people’s horizontal and vertical individualism and collectivism (n = 4260)

Items	Factor loading ^a^
Vertical individualism	
1. It is important that I do my job better than others	0.272
2. Winning is everything	0.525 *
3. Competition is the law of nature	0.513 *
4. When another person does better than I do, I get tense and aroused	0.606 *
Horizontal individualism	
1. I’d rather depend on myself than others	0.726 *
2. I rely on myself most of the time; I rarely rely on others	0.672 *
3. I often do “my own thing.”	0.446 *
4. My personal identity, independent of others, is very important to me	0.262
Vertical collectivism	
1. Parents and children must stay together as much as possible	0.609 *
2. It is my duty to take care of my family, even when I have to sacrifice what I want	0.668 *
3. Family members should stick together, no matter what sacrifices are required	0.550 *
4. It is important to me that I respect the decisions made by my groups	0.807 *
Horizontal collectivism	
1. If a coworker gets a prize, I would feel proud	0.631 *
2. The well-being of my coworkers is important to me	0.635 *
3. To me, pleasure is spending time with others	0.747 *
4. I feel good when I cooperate with others	0.240

^a^Exploratory factor analysis (EFA)

^*^Factor loadings for each scale item indicate onto which subfactor the scale item loaded

#### Political views

Participants’ political views were recorded on a 7-point scale using the question “Please rate your political views on a 7-point scale,” where “1” indicates “very liberal”, “4” means “neutral”, and “7” indicates “very conservative.” For our analysis, we re-coded the raw political view score into three categories: “liberal” (i.e., a response score of 1–3), “neutral” (i.e., a response score of 4), and “conservative” (i.e., a response score of 5–7). Additional file 1: Figure S2 (c) presents the distribution of participants’ political views in the six study areas. Participants from Western study areas (i.e., the U.S., the U.K., and New Zealand) have more diverse political views than participants from Eastern study areas (i.e., Hong Kong, Japan, and South Korea). In other words, most participants from Eastern study areas reported that they are politically neutral. Meanwhile, the proportion of “liberal” participants is similar to “conservative” participants in the U.S., the U.K., and New Zealand. The proportion of liberal participants is higher than conservative participants in Hong Kong, which is the opposite of Japan and South Korea.

#### Perceived social tightness

In addition, this section of the questionnaire includes the 6 items shown in Table 3 from Gelfand et al. [22, 23] that measure the cultural tightness of the six study areas (cultural tightness is the strength of norms in a study area and the tolerance for people who violate norms). For these 6 items, participants also assessed their agreement on these items using a 7-point scale (from 1 to 7), where a higher number stands for a higher level of agreement. Then, we also applied EFA to evaluate and validate the one-factor structure underlying the 6 items. We expected that people’s perceived social tightness scores would be explained by one underlying factor at the individual level. Table 3 presents the items and factor loadings. As predicted, the EFA results demonstrated a clear one-factor solution (eigenvalue = 2.36). A higher score means that the participant perceives the social norms to be tighter and the tolerance for deviance in their society to be lower. Additional file 1: Figure S2 (d) shows the distribution of participants’ perceived social norms in the six study areas. It indicates that participants from Eastern study areas (i.e., Hong Kong, Japan, and South Korea) generally have higher perceived social tightness scores than those from Western study areas (i.e., the U.S., the U.K., and New Zealand).

**Table 3:** Questions and factor loadings for people’s perceived social tightness (n = 4260)

Items	Factor loading ^a^
Perceived social tightness	
1. There are many social norms that people are supposed to abide by in this country	0.514 *
2. In this country, there are very clear expectations for how people should act in most situations	0.744 *
3. People agree upon what behaviors are appropriate versus inappropriate in most situations in this country	0.668 *
4. People in this country have a great deal of freedom in deciding how they want to behave in most situations. (Reverse coded)	0.326 *
5. In this country, if someone acts in an inappropriate way, others will strongly disapprove	0.432 *
6. People in this country almost always comply with social norms	0.455 *

^a^Exploratory factor analysis

^*^Factor loadings for each scale item indicate onto which subfactor the scale item loaded

#### Perceived COVID-19 risk

People perceived COVID-19 risk was assessed through the 3 items shown in Table 4: (1) “The COVID-19 pandemic is still not under control.”; (2) “I do not know the risk of the COVID-19 pandemic.”; (3) “The COVID-19 pandemic is catastrophic.” We solicited the level of participants’ agreement on the three items using a 7-point scale. Then, we also applied EFA to evaluate and validate the one-factor structure underlying the 3 items. We expected that people’s perceived COVID-19 risk would be explained by one factor at the individual level underlying the 3 items. The EFA result demonstrated a clear one-factor solution, and the item loadings were greater than 0.5, except for one reverse-coded item that had a loading of 0.149, which followed the expectation. A higher score on perceived COVID-19 risk means that the participant perceived the COVID-19 pandemic to be more severe. Additional file 1: Figure S2 (e) presents the distribution of participants’ perceived COVID-19 risk in the study areas. It indicates that participants from the U.S., Hong Kong, Japan, and South Korea have higher perceived COVID-19 risk scores than those from the U.K., and New Zealand.

**Table 4:** Questions and factor loadings for people’s perceived COVID-19 risk (n = 4260)

Items	Factor loading ^a^
Perceived COVID-19 risk	
The COVID-19 pandemic is still not under control	0.916 *
I do not know the risk of the COVID-19 pandemic. (Reverse coded)	0.149 *
The COVID-19 pandemic is catastrophic	0.518 *

^a^Exploratory factor analysis

^*^Factor loadings for each scale item indicate onto which subfactor the scale item loaded

### Statistical Analysis

We first conducted descriptive analyses (i.e., the mean values and 95% confidence intervals) to chart how the trends of people’s privacy concerns, perceived social benefits, and acceptance of the ten COVID-19 measures varied due to the amount of IGD they use or disclosed to the general public. Specifically, we described how people’s privacy concerns, perceived social benefits, and acceptance of the COVID-19 measures varied across the three types of control measures (contact tracing, self-quarantine monitoring, and location disclosure).

Second, we examined the effects of people’s privacy concerns and perceived social benefits on their acceptance of the ten COVID-19 control measures. To do so, we fitted three multilevel linear regression models that include people’s privacy concerns (PC) and perceived social benefits (SB) as the key independent variables to predict their acceptance (AP). We first used people’s privacy concerns and perceived social benefits respectively as the predictor variable in Models 1a and 1b. In the full model (Model 1c), both people’s privacy concerns and perceived social benefits are included as the predictor variables. We fitted the multilevel models to simultaneously control for the within and between country/region effects on people’s acceptance. Therefore, all models include a set of individual-level (i.e., people’s age, gender, educational status, COVID-19 infection status, perceived COVID-19 risk) and country-level (i.e., case fatality rate and per capita income) covariates as controls. The three multilevel linear regression models were implemented using ‘lme4’ (v.1.1–31) and ‘lmerTest’ (v.3.1–3) packages in R statistical software (version 4.1). Equation (1) summarizes the models, with *β* denoting the coefficients, *ε* and *π* denoting the random effects of the individual-level and the country-level variables respectively:

1 $$\begin{gathered} AP_{{ij}} = \alpha _{0} + \beta _{1} PC + \beta _{2} SB + \beta _{3} \,Controls + \varepsilon _{{ij}} + \pi _{j} \hfill \\ {\text{with}}\,i\,\left( {{\text{individual}}} \right){\text{ }} = {\text{ 1}},{\text{ }} \ldots ,;\,j\,\left( {{\text{country}}} \right){\text{ }} = {\text{ 1}},{\text{ }} \ldots ,{\text{ 6}} \hfill \\ \end{gathered}$$

To assess the impacts of people’s political views (liberal versus conservative), perceived social tightness (i.e., the strength of social norms), and individualist orientation (both vertical and horizontal individualism are considered) on their privacy concerns (PC), perceived social benefits (SB), and acceptance (AP), we fitted a series of multilevel linear regression models. In the first set of models (Models 2a, 3a and 4a), we used people’s political views (PV) as the predictor variable. In the next step (Models 2b, 3b and 4b), people’s political views (PV) and perceived social tightness (ST) were included. The third set of models (Models 2c, 3c and 4c) included people’s political views (PV), perceived social tightness (ST), vertical individualism (VI), and horizontal individualism (HI). We further included people’s privacy as a key independent variable in Model 3d to predict their perceived social benefits. Meanwhile, both people’s privacy concerns and perceived social benefits were included in Model 4d as the key predictor variables for people’s acceptance. All models also included the same individual-level and country-level covariates as controls. We also used ‘lme4’ (v.1.1–31) and ‘lmerTest’ (v.3.1–3) packages in R statistical software (version 4.1) to fit all multilevel linear regression models. Equations (2), (3), and (4) summarize the models, with *β* denoting the coefficients, *ε* and *π* denoting the random effects of the individual-level and the country-level covariates respectively:

2 $${PC}_{ij}={\alpha }_{0}+{\beta }_{1}PV+{\beta }_{2}ST+{\beta }_{3}VI+{\beta }_{4}HI+{\beta }_{5}Controls+{\varepsilon }_{ij}+{\pi }_{j}$$

3 $${SB}_{ij}={\alpha }_{0}+{\beta }_{1}PV+{\beta }_{2}ST+{\beta }_{3}VI+{\beta }_{4}HI+{\beta }_{5}PC+{\beta }_{6}Controls+{\varepsilon }_{ij}+{\pi }_{j}$$

4 $$\begin{gathered} AP_{ij} = \alpha_{0} + \beta_{1} PV + \beta_{2} ST + \beta_{3} VI + \beta_{4} HI + \beta_{5} PC + \beta_{6} SB + \beta_{7} Controls + \varepsilon_{ij} + \pi_{j} \hfill \\ {\text{with}}i\left( {{\text{individual}}} \right) \, = { 1}, \, \ldots ,;j\left( {{\text{country}}} \right) \, = { 1}, \, \ldots ,{ 6} \hfill \\ \end{gathered}$$

Finally, we fitted three multilevel structure equation models (SEMs) to explore the direct, indirect, and mediating effects among the variables. The multilevel SEMs were designed to evaluate and validate the robustness of the abovementioned multilevel linear regression models. Therefore, the first model (Model 5a) tested the following hypothesis: (1) People’s political views, perceived social tightness, and individualism have direct effects on their privacy concerns, perceived social benefits, and acceptance of the COVID-19 control measures. The second model (Model 5b) focused on testing hypothesis (1) and the second hypothesis: (2) People’s privacy concerns and perceived social benefits play mediating roles in the effects of people’s political views, individualism, and perceived social tightness on their acceptance of COVID-19 control measures. The third model (Model 5c) further tested hypotheses (1) and (2) and the third hypothesis: (3) People’s perceived social benefits play a mediating role in the effects of people’s privacy concerns on their acceptance of COVID-19 control measures. The path diagrams of the estimated models are presented in Additional file 1: Figures S3-S5. The three SEMs were implemented with MPlus (version 8.3).

## Results

### The associations between people’s acceptance with perceived social benefits and privacy concerns

**Fig. 2:**
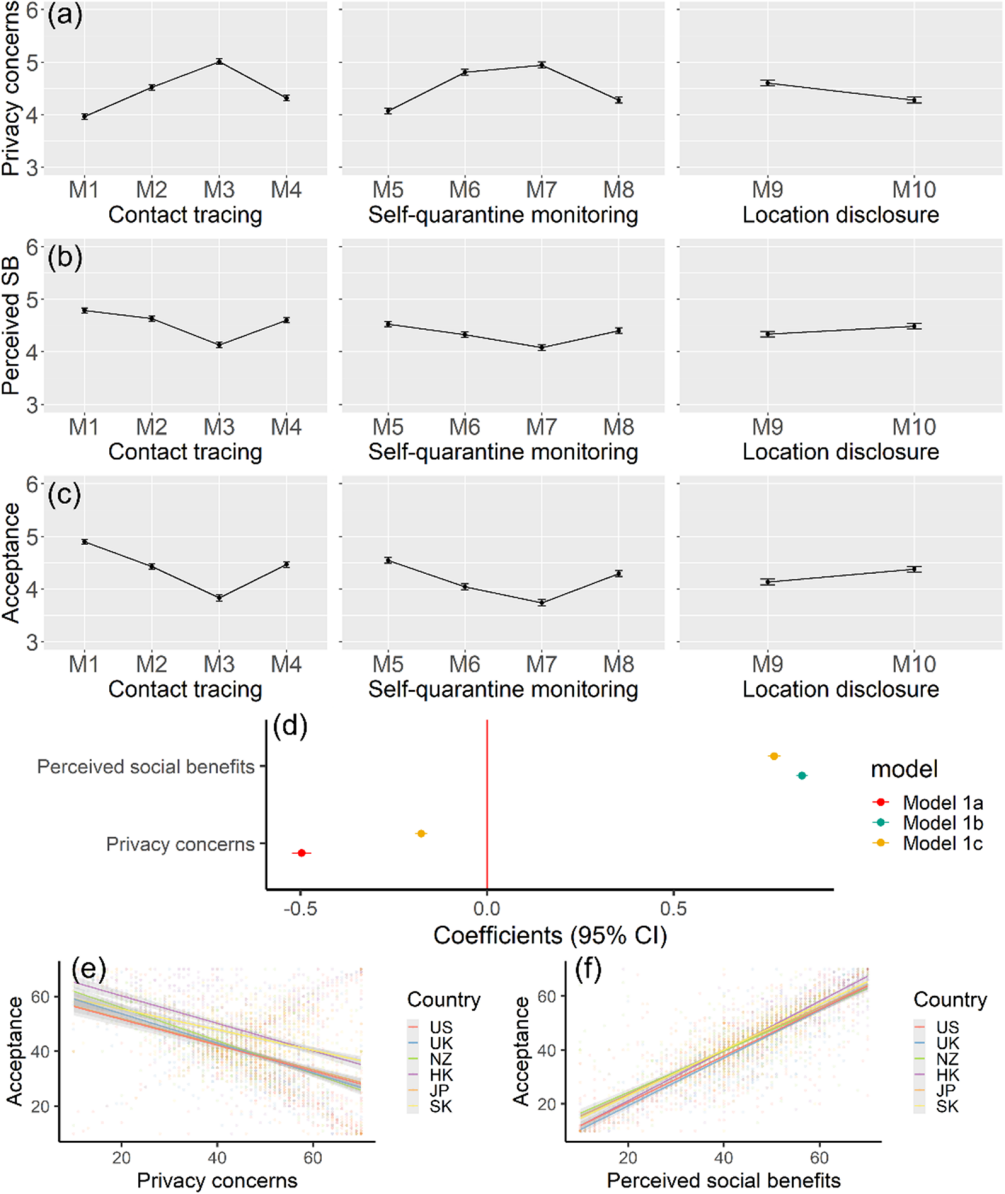
Results of statistical description of the ten COVID-19 control measures, and multilevel linear regression analysis with acceptance as the dependent variable, and privacy concerns and perceived social benefits as the independent variables: **a**–**c** Mean values and 95% CI of privacy concerns, perceived social benefits, and acceptance; **d** estimated coefficients with 95% CI in different models after controlling for individual-level and country or region-level covariates; **e** linear relationship between acceptance and privacy concerns in the six study areas; **f** linear relationship between acceptance and perceived social benefits in the six study areas

The mean values and 95% confidence intervals of people’s privacy concerns, perceived social benefits, and acceptance of the ten COVID-19 measures are shown in Fig. 2a–c. The results suggest that people have higher levels of privacy concerns and lower levels of perceived social benefits and acceptance of COVID-19 control measures that use or disclose more individual-level location information. Specifically, among the contact tracing and self-quarantine measures (i.e., M1–M8), people have higher levels of privacy concerns and lower levels of perceived social benefits and acceptance for the measures that obtain their location information from mobile phones and credit card histories or require them to wear an e-wristband (i.e., M2, M3, M6, and M7) than other measures (e.g., obtain people’s location by conducting conventional interviews or random calls). Besides, people also have higher levels of privacy concerns and lower levels of perceived social benefits and acceptance if government authorities publicly disclose more individual-level information (e.g., see the comparison between M9 and M10).

We used multilevel linear regression models to examine the effects of people’s privacy concerns and perceived social benefits on the acceptance of COVID-19 control measures. Additional file 1: Table S2 presents the descriptive statistics for individual-level and country-level variables. Figure 2d shows the model results, which indicate a substantial relationship between people’s acceptance of COVID-19 control measures and their privacy concerns and perceived social benefits: higher levels of privacy concerns are associated with lower levels of acceptance, while higher levels of perceived social benefits are consistently associated with higher levels of acceptance. As Models 1a and 1b in Additional file 1: Table S3 reported, people’s privacy concerns (Coef. = -0.50, *p*-value < 0.001, Model 1a) and perceived social benefits (Coef. = 0.84, *p*-value < 0.001, Model 1b) are important predictors of their acceptance of COVID-19 control measures. Model 1c in Additional file 1: Table S3 further suggests that people’s perceived social benefits play a more important role than their privacy concerns in the acceptance of COVID-19 control measures since the negative coefficient of privacy concerns dropped from -0.50 to -0.18 when both predictors (privacy concerns and perceived social benefits) are considered. Figure 2e and f indicate that the effects of people’s privacy concerns and perceived social benefits on their acceptance are consistent across the six study areas.

### The associations between people’s political views, perceived social norms, and individualism with their privacy concerns, perceived social benefits, and acceptance

**Fig. 3:**
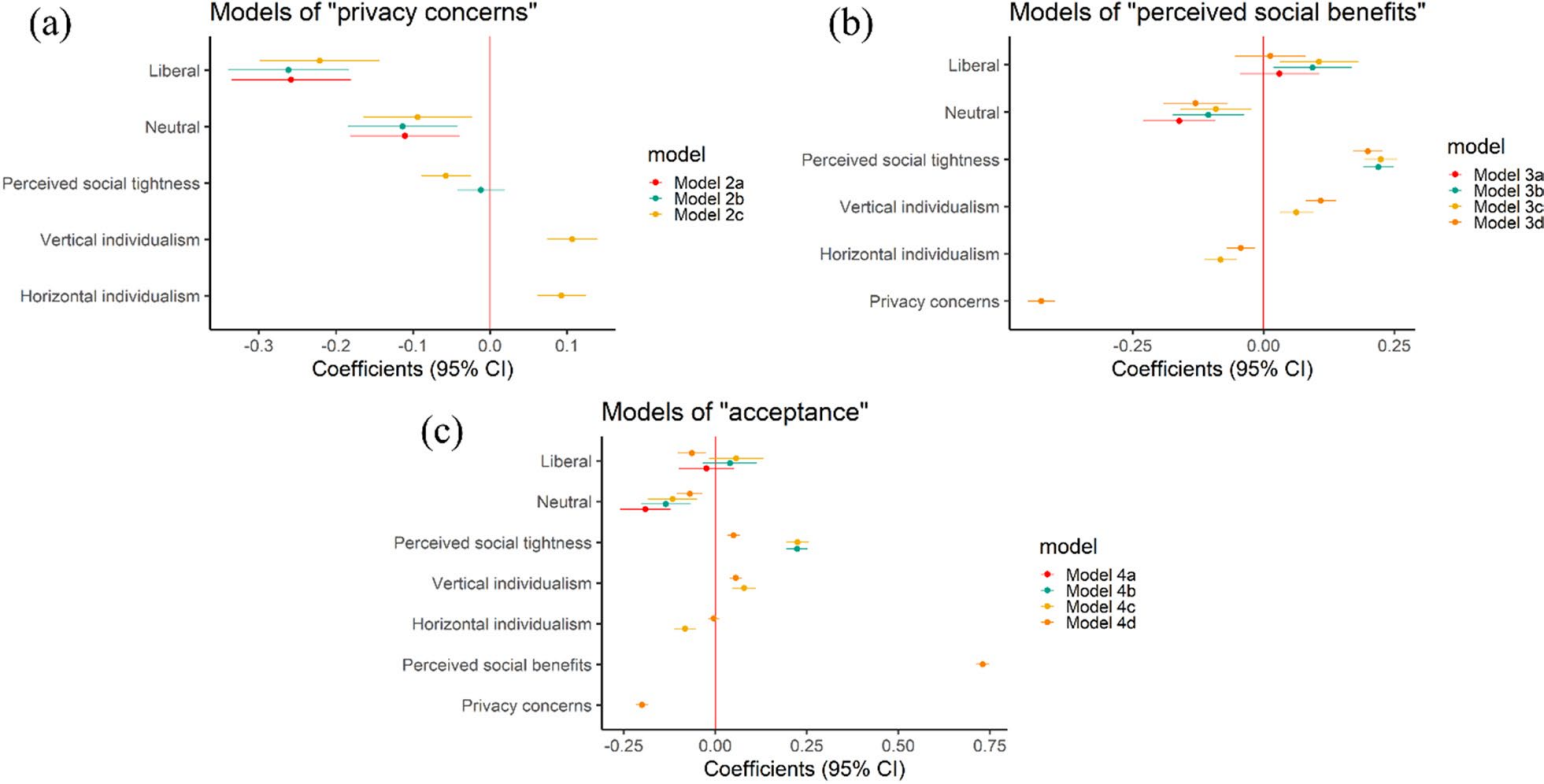
Results of multilevel linear regression analysis with privacy concerns, perceived social benefits, and acceptance as the dependent variables, and political views, perceived social tightness, vertical individualism, and horizontal individualism as the independent variables. **a** estimated coefficients with 95% CI in different models with privacy concerns as the dependent variable; **b** estimated coefficients with 95% CI in different models with perceived social benefits as the dependent variable; **c** estimated coefficients with 95% CI in different models with acceptance as the dependent variable. All models are controlled for individual-level and country or region level covariates. (The results are from models presented in Additional file 1: Tables S4–S6)

We further used multilevel linear regression models to assess the importance of people’s individualist orientation (both vertical and horizontal individualism), political views (liberal versus conservative), and perceived social tightness (i.e., the strength of social norms) in their privacy concerns, perceived social benefits, and acceptance of COVID-19 control measures. As shown in Fig. 3a, people’s political views, vertical individualism, and horizontal individualism are significantly and consistently associated with their privacy concerns. As shown in Additional file 1: Table S4, people who have a liberal political view (Coef. = − 0.22, *p*-value < 0.001, Model 2c) and a neutral political view (Coef. = − 0.09, *p*-value < 0.01, Model 2c) tend to have a lower level of privacy concerns when compared to those who have a conservative political view. People’s vertical (Coef. = 0.11, *p*-value < 0.001, Model 2c) and horizontal individualism (Coef. = 0.09, *p*-value < 0.001, Model 2c) are consistently positively associated with their privacy concerns. Meanwhile, we observed a weak negative association between people’s privacy concerns and perceived social tightness (Coef. = -0.06, *p*-value < 0.001, Model 2c) after simultaneously including people’s political views, perceived social tightness, vertical individualism, and horizontal individualism in the full model (Model 2c).

Figure 3b and c show people’s perceived social benefits and acceptance are consistently significantly associated with their perceived social tightness, vertical individualism and horizontal individualism. There is no consistent relationship between people’s perceived social benefits and acceptance with their political views (i.e., conservative vs liberal). Additional file 1: Tables S5 and S6 present the results of the models. First, we observed that people’s perceived social tightness has consistently positive associations with their perceived social benefits (Coef. = 0.20–0.22, p-value < 0.001, Models 3b–3d) and acceptance (Coef. = 0.22, p-value < 0.001, Models 4b and 4c). The results also reveal that people’s vertical individualism has consistently positive associations with their perceived social benefits (Coef. = 0.06–0.11, p-value < 0.001, Model 3c–3d) and acceptance (Coef. = 0.06–0.08, p-value < 0.001, Models 4c and 4d), while horizontal individualism has negative associations with their perceived social benefits (Coef. = − 0.04 to − 0.08, p-value < 0.001, Models 3c–3d) and acceptance (Coef. =  − 0.08, p-value < 0.001, Model 4c). These associations for people’s perceived social benefits are still valid after including people’s privacy concerns as a predictor variable in the full model (Model 3d). However, the effects of people’s perceived social tightness and horizontal individualism on acceptance become weak after including people’s privacy concerns and perceived social benefits as the predictor variables in the full model (Model 4d): the coefficient of people’s perceived social tightness dropped from 0.22 to 0.05, and horizontal individualism become insignificant associated with acceptance.

**Fig. 4:**
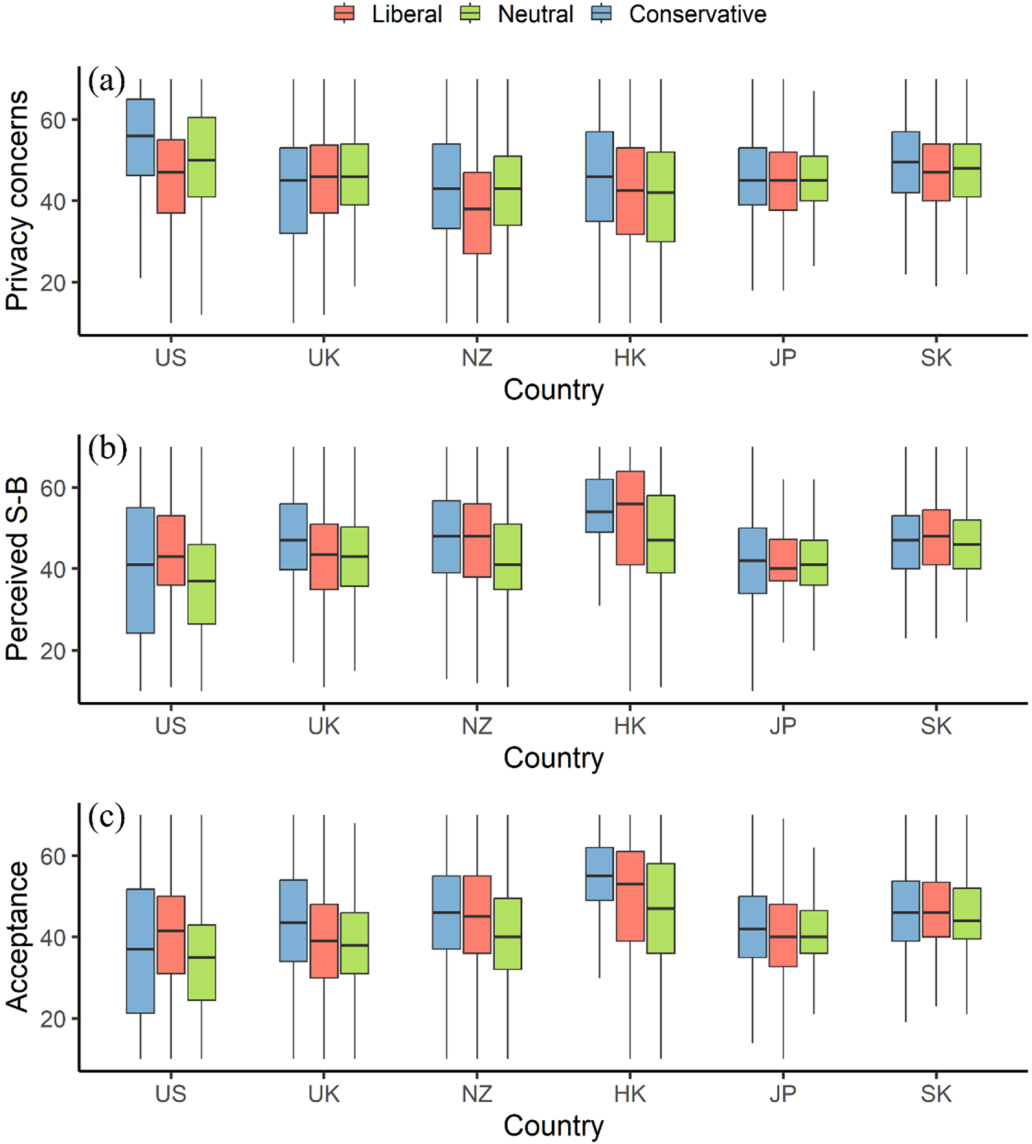
The effect of political views (i.e., Liberal vs Neutral vs Conservative) in people’s privacy concerns, perceived social benefits, and acceptance by study areas: **a** privacy concerns; **b** perceived social benefits (SB); **c** acceptance

Figure 4 shows the heterogeneous effects of people’s political views on their privacy concerns, perceived social benefits, and acceptance across the six study areas. Figure 4a reported that the level of privacy concerns decreased from conservatives to liberals for people living in the U.S., New Zealand, Hong Kong, and South Korea. Besides, people living in the U.S. with different political views reported the largest difference in privacy concerns. However, Fig. 4b and c suggested that the effects of political views on people’s perceived social benefits and acceptance are mixed across the six study areas.

**Fig. 5:**
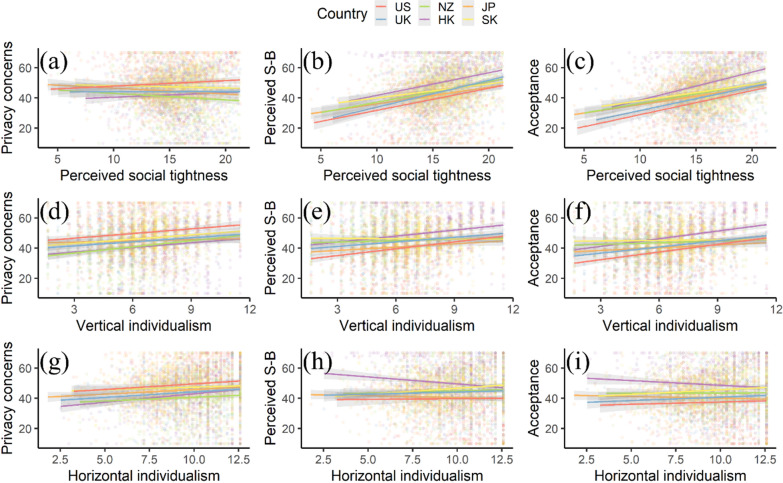
Linear relationships between the predictor variables with people’s privacy concerns, perceived social benefits, and acceptance across the study areas: **a**–**c** the linear relationships between people’s perceived social tightness with their privacy concerns, perceived social benefits, and acceptance; **d**–**f** the linear relationships between people’s vertical individualism with their privacy concerns, perceived social benefits, and acceptance; **g**–**i** the linear relationships between people’s horizontal individualism with their privacy concerns, perceived social benefits, and acceptance

Figure 5 shows linear relationships between people’s perceived social tightness, vertical individualism, and horizontal individualism with their privacy concerns, perceived social benefits, and acceptance across the six study areas. Figure 5a presents a valid and tiny negative association between people’s privacy concerns and perceived social tightness in the U.K., New Zealand, Japan, and South Korea. Figures 5b and c show consistently significant positive relationships between people’s perceived social benefits and acceptance with their perceived social tightness across the six study areas. Figure 5d–f present clear positive relationships between people’s vertical individualism with their privacy concerns, perceived social benefits, and acceptance in the study areas. As shown in Fig. 5g, horizontal individualism has a consistently significant positive relationship with privacy concerns, while Fig. 5h and i show that it has mixed relationships with perceived social benefits and acceptance across the six study areas.

### Multilevel structure equation models result

**Fig. 6:**
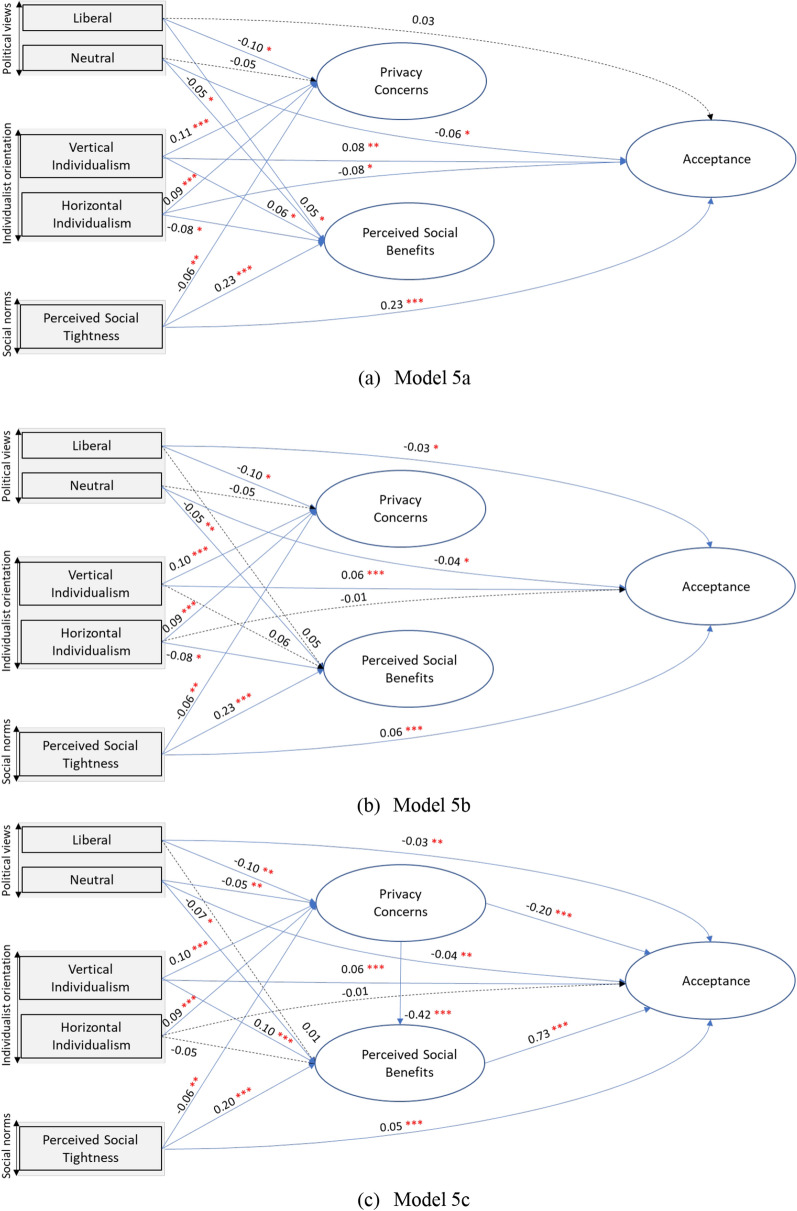
The path diagrams of the results for the multilevel structural equation models (SEMs), a blue solid arrow with one (p < 0.05), two (p < 0.01), or three (p < 0.001) red asterisks (*) indicates a significant path coefficient between the variables. A black dashed arrow indicates a non-significant path coefficient between the variables. **a** Model 5a examines hypothesis 1; **b** Model 5b examines hypotheses 1 and 2; **c** Model 5c examines hypotheses 1–3

We also replicated the above results of the multilevel linear regression models using three multilevel structure equations models (SEMs). The path diagram of the estimated models and results are presented in Fig. 6 and Additional file 1: Tables S7–S9. All SEMs fit well, and the results are similar to those obtained with the multilevel linear regression models reported in the results section. Specifically, we also observed that people who have a liberal political view and a neutral political view tend to have a lower level of privacy concerns when compared to those who have a conservative political view, and the effects of people’s political views on their perceived social benefits and acceptance are weak. People’s perceived social tightness has strong and direct impacts on their perceived social benefits (Coef. = 0.20–0.23, p-value < 0.001, Models 5a-5c) and acceptance (Coef. = 0.23, p-value < 0.001, Model 5a). However, the direct impacts of people’s perceived social tightness on their acceptance become weak (i.e., the coefficient dropped from 0.23 in Model 5a to 0.06 and 0.05 in Models 5b and 5c) after including the mediating roles of privacy concerns and perceived social benefits in the models. People’s vertical individualism has consistent direct positive impacts on their privacy concerns, perceived social benefits, and acceptance. Meanwhile, people’s horizontal individualism also has consistent direct positive impacts on their privacy concerns. We observed direct negative impacts of horizontal individualism on perceived social benefits (Coef. = − 0.08, p-value < 0.05) and acceptance (Coef. = − 0.08, p-value < 0.05) in Model 5a, and the impacts become weak (i.e., the coefficient dropped from − 0.08 with p-value < 0.05 in Model 5a to − 0.01 p-value > 0.05 in Models 5b and 5c) in models that include the mediating roles of privacy concerns and perceived social benefits. As expected, the results also indicated the direct negative impacts of privacy concerns (Coef. =  − 0.20to − 0.22, p-value < 0.001, Models 5b and 5c) and perceived social benefits (Coef. = 0.73–0.78, p-value < 0.001, Models 5b and 5c) on their acceptance, and a direct negative impact of people’s privacy concerns (Coef. =  − 0.42, p-value < 0.001, Model 5c) on their perceived social benefits.

## Conclusions and discussion

The results of this study confirm the importance of people’s privacy concerns and perceived social benefits in explaining the differences in their acceptance of COVID-19 control measures across the study areas. Besides, the results also suggested that people’s views of the control measures (i.e., privacy concerns, perceived social benefits, and acceptance) were strongly associated with their political views, perceived social tightness, and individualist orientation. The associations remain consistent in the six study countries and areas, although they might be weaker or stronger in certain countries. Therefore, our findings could be generalized to other Western and Eastern countries. These results indicate the importance of the social and cultural context in shaping the relationships between people’s privacy concerns, perceived social benefits, and acceptance of the control measures.

Our findings provide critical insights into people’s acceptance of COVID-19 control measures and how public health agencies can better address the trade-off between disease control and geoprivacy protection. As our results indicate, people’s concerns about the control measures for violating geoprivacy significantly affect their perceived social benefits and acceptance of these measures, which in turn undermines the effectiveness of these measures in controlling the spread of COVID-19 or future pandemics. At the individual level, there is a tradeoff relationship between people’s privacy concerns and the perceived social benefits of the control measures, which in turn determines the extent to which people would accept a control measure (i.e., by giving up some of their privacy and receiving the benefits of pandemic control in return). Thus, to effectively control a pandemic or highly contagious infectious disease like COVID-19, it is important for governments to carefully achieve a socially acceptable balance between disease control and geoprivacy protection. It is also important because geoprivacy breaches may bring forth serious unintended negative consequences such as the stigmatization of and discrimination against infected persons. Further, as our study also indicates, people have lower levels of acceptance for COVID-19 control measures that disclose more individual-level location information. Such knowledge can help governments and public health agencies to identify and implement measures that would be more acceptable to people in specific social and cultural contexts.

Besides, our findings indicate that differences in people’s political views, perceived social tightness, and individualist orientation across the study areas also play important roles in the different levels of acceptance of COVID-19 control measures that use IGD. People with liberal and neutral political views tend to have lower levels of privacy concerns than people with conservative political views. In the U.S., people with different political views reported the largest difference in privacy concerns. By examining the effects of people’s political views on their health behaviors (e.g., geoprivacy concerns of COVID-19 control measures and its relationship with people’s privacy concerns), our study also adds to the recent literature that observed how liberals and conservatives differ in various aspects of health behaviors in the U.S. during the COVID-19 pandemic [24–26].

Our findings showed that people’s perceived social tightness is consistently positively associated with their acceptance and perceived social benefits of COVID-19 control measures across the study areas. This is consistent with previous studies, which revealed that people in societies with tighter social norms tend to be more cooperative when compared to people in societies with looser social norms during the COVID-19 pandemic, and thus countries with tighter social norms tend to achieve better performance in controlling COVID-19 than countries with looser social norms [22]. However, our results further indicated that the impacts of people’s perceived social tightness on acceptance would become weak after considering the moderating role of people’s privacy concerns. Besides, we did not find consistent relationships between people’s perceived social tightness and privacy concerns across the study areas. These results suggest that strengthening social norms might improve people’s perceived social benefits and thus increase their acceptance of COVID-19 control measures but cannot reduce their privacy concerns. This study further contributes to the examination of the effects of social norms on public health crises and is among the first to consider the role of individuals’ privacy concerns.

Going beyond previous studies, this study considered the influences of different dimensions of individualism on people’s perceived social benefits and acceptance of COVID-19 control measures. While the importance of people’s individualistic orientation for COVID-19 control has been examined in recent studies [27–30], most of these studies overlooked the increase in the levels of individualism across the world over the past few decades, a non-negligible trend in both Western and Eastern regions and the world at large [31–33]. Instead of comparing the effects of people’s individualistic orientation with the effects of people’s collectivistic orientation, we highlight the differences in people’s individualistic tendencies via the vertical and horizontal dimensions, where vertical individualism stresses competition among social groups, while horizontal individualism stresses the uniqueness of individuals [20]. Our findings first suggest consistent direct negative impacts of people’s vertical and horizontal individualism on their privacy concerns. Then, the results further uncovered opposite relationships between people’s acceptance and perceived social benefits of COVID-19 control measures with their vertical and horizontal individualism: vertical individualism has a positive association with acceptance and perceived social benefits, while horizontal individualism has a significant negative one. However, the impacts of people’s horizontal individualism on their acceptance and perceived social benefits are weaker than vertical individualism. Vertical individualists stress individuals’ own responsibility and tend to attribute success to themselves [20, 34], and thus they are more likely to think that their individual acceptance of COVID-19 control measures have essential consequences for social benefits during the pandemic.

Consistent with previous studies [22], our results suggest that strengthening people’s perceived social norms might improve people’s acceptance and perceived social benefits of COVID-19 control measures, which would help prevent disease transmission during the COVID-19 pandemic. However, our results also indicate that strengthening social norms has a weak impact on people’s acceptance, and it cannot reduce people’s privacy concerns, which might undermine people’s sustained acceptance of pandemic control measures and can be a barrier to the prevention of disease transmission. Moreover, people’s privacy concerns tend to increase with their conservatism and individualism. Note that increasing evidence indicates that conservative groups are gaining influence in different countries (e.g., Brazil, France, Poland, Austria, Italy, Hungary, and Sweden) around the world [35–37], and there is an upward trend in the levels of individualism across the world over the past few decades. Therefore, our results also imply the following conclusion: people’s privacy concerns will keep rising with the global conservatism and individualism trends, and this would be a significant factor that influences the effectiveness of future efforts of pandemic control. However, because we observed that vertical individualism has positive associations with acceptance and perceived social benefits of COVID-19 control measures, our results also highlight the importance of building stronger connections among individuals for controlling future pandemics.

Our study provides strong evidence that supports the potential causal pathways through which people’s political views, perceived social norms, and individualism shape their privacy concerns for and acceptance of COVID-19 control measures that use IGD. However, our cross-sectional data do not facilitate causal inference, although we have considered within-country/area variations in the multilevel models, including controlling for important individual-level (i.e., people’s age, gender, educational level, COVID-19 infection status, perceived pandemic risk) and country-level (i.e., case fatality rate and per capita income) covariates. Future studies should further examine whether the important findings revealed in this study are also valid for the same individuals in different periods using a longitudinal study design. We also observed the largest effect of political views on people’s views of COVID-19 control measures in the U.S. Some important plausible reasons can be the highly polarized politics in the country: the differences between individuals’ health behaviors are highly associated with their partisanship (i.e., Democrat or Republican) [24, 38, 39]. For instance, some cues and tips provided by past-president Trump stimulated many Republican voters to oppose the social distancing directives issued by the Centers for Disease Control and Prevention during the early stage of the pandemic [40]. Therefore, future research could explore whether party leaders could mitigate the differences between people’s privacy concerns due to their political views.

### Supplementary Information

**Additional file 1: Table S1.** A detailed description of the ten COVID-19 control measures in the survey. **Table S2.** Descriptive statistics for individual-level and country-level variables (n=4260). **Table S3.** Multilevel linear regression models examining the relationships between people’s acceptance and their privacy concerns and perceived social benefits (n=4260). **Table S4.** Multilevel linear regression models examining the relationships between people’s privacy concerns and their political views, perceived social tightness, vertical and horizontal individualism (n=4260). **Table S5.** Multilevel linear regression models examining the relationships between people’s perceived social benefits and their political views, perceived social tightness, vertical and horizontal individualism, and privacy concerns (n=4260). **Table S6.** Multilevel linear regression models examining the relationships between people’s acceptance and their political views, perceived social tightness, vertical and horizontal individualism, privacy concerns, and perceived social benefits (n=4260). **Table S7.** Multilevel structural equation model examining hypothesis: (1) people’s political views, perceived social tightness, and individualism have direct effects on their privacy concerns, perceived social benefits, and acceptance of the COVID-19 control measures (n=4260). **Table S8.** Multilevel structure equation model examining hypotheses: (1) people’s political views, perceived social tightness, and individualism have direct effects on their privacy concerns, perceived social benefits, and acceptance of the COVID-19 control measures; (2) people’s privacy concerns and perceived social benefits play mediating roles in the effects of people’s political views, individualism, and perceived social tightness on their acceptance of COVID-19 control measures (n=4260). **Table S9.** Multilevel structure equation model examining hypotheses: (1) people’s political views, perceived social tightness, and individualism have direct effects on their privacy concerns, perceived social benefits, and acceptance of the COVID-19 control measures; (2) people’s privacy concerns and perceived social benefits play mediating roles in the effects of people’s political views, individualism, and perceived social tightness on their acceptance of COVID-19 control measures; (3) people’s perceived social benefits play a mediating role in the effects of people’s privacy concerns on their acceptance of COVID-19 control measures (n=4260). **Figure S1.** Statistical distributions of people’s views on the COVID-19 control measures in the six study areas. (a) Privacy concerns; (b) Perceived social benefits (S-B); and (c) Acceptance. **Figure S2.** Statistical distributions of people’s vertical and horizontal individualism, political views, perceived social tightness, and perceived COVID-19 risk across the six study areas. (a) vertical individualism; (b) horizontal individualism; (c) political views; (d) perceived social tightness; (e) perceived COVID-19 risk. **Figure S3.** Conceptual model (Model 5a) for examining the hypothesis: (1) people’s political views, perceived social tightness, and individualism have direct effects on their privacy concerns, perceived social benefits, and acceptance of the COVID-19 control measures. **Figure S4.** Conceptual model (Model 5b) for examining the hypotheses: (1) people’s political views, perceived social tightness, and individualism have direct effects on their privacy concerns, perceived social benefits, and acceptance of the COVID-19 control measures; (2) people’s privacy concerns and perceived social benefits play mediating roles in the effects of people’s political views, individualism, and perceived social tightness on their acceptance of COVID-19 control measures. **Figure S5.** Conceptual model (Model 5c) for examining the hypotheses: (1) people’s political views, perceived social tightness, and individualism have direct effects on their privacy concerns, perceived social benefits, and acceptance of the COVID-19 control measures; (2) people’s privacy concerns and perceived social benefits play mediating roles in the effects of people’s political views, individualism, and perceived social tightness on their acceptance of COVID-19 control measures; (3) people’s perceived social benefits play a mediating role in the effects of people’s privacy concerns on their acceptance of COVID-19 control measures.

## Data Availability

The dataset will be available upon request. Please contact the corresponding author M.P.K.
