# Supplementary material for: People’s political views, perceived social norms, and individualism shape their privacy concerns for and acceptance of pandemic control measures that use individual-level georeferenced data
Source: Int J Health Geogr. 2023 Dec 6;22:35. doi: 10.1186/s12942-023-00354-3 (PMC10702027; doi:10.1186/s12942-023-00354-3)
Supplement: Supplementary file 1 — Additional file 1: Table S1. A detailed description of the ten COVID-19 control measures in the survey. Table S2. Descriptive statistics for individual-level and country-level variables (n=4260). Table S3. Multilevel linear regression models examining the relationships between people’s acceptance and their privacy concerns and perceived social benefits (n=4260). Table S4. Multilevel linear regression models examining the relationships between people’s privacy concerns and their political views, perceived social tightness, vertical and horizontal individualism (n=4260). Table S5. Multilevel linear regression models examining the relationships between people’s perceived social benefits and their political views, perceived social tightness, vertical and horizontal individualism, and privacy concerns (n=4260). Table S6. Multilevel linear regression models examining the relationships between people’s acceptance and their political views, perceived social tightness, vertical and horizontal individualism, privacy concerns, and perceived social benefits (n=4260). Table S7. Multilevel structural equation model examining hypothesis: (1) people’s political views, perceived social tightness, and individualism have direct effects on their privacy concerns, perceived social benefits, and acceptance of the COVID-19 control measures (n=4260). Table S8. Multilevel structure equation model examining hypotheses: (1) people’s political views, perceived social tightness, and individualism have direct effects on their privacy concerns, perceived social benefits, and acceptance of the COVID-19 control measures; (2) people’s privacy concerns and perceived social benefits play mediating roles in the effects of people’s political views, individualism, and perceived social tightness on their acceptance of COVID-19 control measures (n=4260). Table S9. Multilevel structure equation model examining hypotheses: (1) people’s political views, perceived social tightness, and individualism have direc [file 12942_2023_354_MOESM1_ESM.docx]

Additional files for

People’s political views, perceived social norms, and individualism shape their privacy concerns for and acceptance of pandemic control measures that use individual-level georeferenced data

# **Additional tables and figures**

Table S1. A detailed description of the ten COVID-19 control measures in the survey.

| **Method** | **Type** | **Description** |
| --- | --- | --- |
|  |  |  |
| M1 | Contact  tracing | Obtaining location information by conducting conventional  interviews |
| M2 |  | Obtaining location information from patients’ mobile phones (e.g., GPS trajectories) |
| M3 |  | Obtaining location information from patients’ credit card  history |
| M4 |  | Bluetooth-based proximity tracing method |
| M5 | Self-Quarantine Monitoring | Monitoring people’s self-quarantine by calling them at random times of day |
| M6 |  | Monitoring people’s self-quarantine by obtaining their real-time locations from their mobile phones (e.g., signal) |
| M7 |  | Monitoring people’s self-quarantine by requiring them to wear an e-wristband that reported their real-time locations to public health officers |
| M8 |  | People were required to carry a valid travel certificate (i.e., not in self-quarantine) when using public places |
| M9 | Location  Disclosure | Publicly disclosing the locations of major activities of COVID-19 patients with their ages and genders |
| M10 |  | Publicly disclosing the locations of major activities of COVID-19 patients (not disclosing ages and genders) |

Table S2. Descriptive statistics for individual-level and country-level variables (n=4260).

| Variables | Percent/Mean ^a^ | Std. |  |
| --- | --- | --- | --- |
| *Age group:* |  |  |  |
| 18-24 years old | 8.10% |  |  |
| 25-44 years old | 44.25% |  |  |
| 45+ years old | 47.65% |  |  |
| *Gender:* |  |  |  |
| Male | 48.54% |  |  |
| Female and others | 51.46% |  |  |
| *Education status:* |  |  |  |
| People with higher education degree | 66.71% |  |  |
| People without higher education degree | 33.29% |  |  |
| *COVID-19 infection:* |  |  |  |
| Infected with COVID-19 (self) | 36.92% |  |  |
| Infected with COVID-19 (family members) | 73.71% |  |  |
| Acceptance | 42.74 | (13.99) |  |
| Privacy concerns | 44.79 | (13.82) |  |
| Perceived social benefits | 44.31 | (13.84) |  |
| *Political views:* |  |  |  |
| Liberal | 27.44% |  |  |
| Neutral | 43.10% |  |  |
| Conservative | 29.46% |  |  |
| Perceived social tightness | 13.94 | (2.44) |  |
| *Individualist orientation:* |  |  |  |
| Vertical individualism | 6.50 | (1.89) |  |
| Horizontal individualism | 9.51 | (1.91) |  |
| Perceived COVID-19 risk | 7.16 | (2.18) |  |
| Country-based variables | | | |
| Country | Case fatality rate ^c^ | Per capita income (USD) ^d^ |  |
| US | 1.1% | 70,248.63 |  |
| UK | 0.9% | 46,510.28 |  |
| NZ | 0.1% | 48,781.03 |  |
| HK | 0.4% | 49,800.54 |  |
| JP | 0.2% | 39,312.66 |  |
| SK | 0.1% | 34,997.78 |  |
| Notes: Standard deviations in round brackets. ^a.^ The descriptive summaries show percentage distributions for categorical variables and mean values for continuous variables; ^b.^ 1=yes, 0=no; ^c.^ World Health Organization data; d. 2021 World Bank data. | | | |

Table S3. Multilevel linear regression models examining the relationships between people’s acceptance and their privacy concerns and perceived social benefits (n=4260).

| Acceptance | | | | |
| --- | --- | --- | --- | --- |
|  |  | Model 1a | Model 1b | Model 1c |
|  |  | Coef.  (S.E.)  [*p*-value] | Coef.  (S.E.)  [*p*-value] | Coef.  (S.E.)  [*p*-value] |
| Age group  (ref. 25 – 44 years old) | 18 - 24 years old | -0.07  (0.05)  [0.170] | -0.01  (0.03)  [0.766] | -0.02  (0.03)  [0.421] |
|  | 45+ years old | 0.04  (0.03)  [0.194] | -0.01  (0.02)  [0.656] | -0.04 *  (0.02)  [0.017] |
| Gender (ref. male) | Female | -0.03  (0.03)  [0.256] | -0.01  (0.02)  [0.693] | -0.01  (0.02)  [0.789] |
| Educational status  (ref. without a high educational degree) | High education | 0.06 *  (0.03)  [0.023] | -0.03  (0.02)  [0.130] | -0.01  (0.02)  [0.522] |
| COVID-19 infection  (ref. never been infected) | Infected with COVID-19  (self) | 0.01  (0.03)  [0.801] | -0.04 *  (0.02)  [0.025] | -0.03  (0.02)  [0.151] |
|  | Infected with COVID-19  (family members) | -0.23 ***  (0.03)  [0.000] | -0.07 ***  (0.02)  [0.000] | -0.11 ***  (0.02)  [0.000] |
|  | **Privacy concerns** | -0.50 ***  (0.01)  [0.000] | - | -0.18 ***  (0.01)  [0.000] |
|  | **Perceived social benefits** | - | 0.84 ***  (0.01)  [0.000] | 0.77 ***  (0.01)  [0.000] |
| Other country-level control variables | Country-level case fatality rate | -0.10  (0.31)  [0.794] | -0.10  (0.07)  [0.482] | -0.09  (0.06)  [0.791] |
|  | Country-level per capita income | 0.19  (0.30)  [0.575] | 0.05  (0.06)  [0.574] | 0.04  (0.05)  [0.837] |
| Intercept |  | 0.26  (0.15)  [0.195] | 0.11 *  (0.03)  [0.021] | 0.14 **  (0.04)  [0.008] |
| Observations |  | 4260 | 4260 | 4260 |
| AIC |  | 10593.38 | 6457.31 | 6057.50 |
| Pseudo-R² (fixed effects) | | 0.23 | 0.73 | 0.76 |
| Pseudo-R² (total) |  | 0.43 | 0.74 | 0.76 |
| Notes: Standard errors are in round brackets and *p*-values are in square brackets; *** denotes p < 0.001. ** denotes p < 0.01. * denotes p < 0.05. | | | | |

Table S4. Multilevel linear regression models examining the relationships between people’s privacy concerns and their political views, perceived social tightness, vertical and horizontal individualism (n=4260).

| Privacy concerns | | | | |
| --- | --- | --- | --- | --- |
|  |  | Model 2a | Model 2b | Model 2c |
|  |  | Coef.  (S.E.)  [*p*-value] | Coef.  (S.E.)  [*p*-value] | Coef.  (S.E.)  [*p*-value] |
| Age group  (ref. 25 – 44 years old) | 18 - 24 years old | -0.05  (0.06)  [0.355] | -0.05  (0.06)  [0.368] | -0.04  (0.06)  [0.487] |
|  | 45+ years old | -0.27 ***  (0.03)  [0.000] | -0.27 ***  (0.03)  [0.000] | -0.25 ***  (0.03)  [0.000] |
| Gender (ref. male) | Female | 0.05  (0.03)  [0.140] | 0.04  (0.03)  [0.143] | 0.09 **  (0.03)  [0.003] |
| Educational status  (ref. without a high educational degree) | High education | 0.08 *  (0.03)  [0.014] | 0.08 *  (0.03)  [0.013] | 0.08 *  (0.03)  [0.020] |
| COVID-19 infection  (ref. never been infected) | Infected with COVID-19  (self) | 0.08 *  (0.03)  [0.012] | 0.08 *  (0.03)  [0.013] | 0.09 **  (0.03)  [0.006] |
|  | Infected with COVID-19  (family members) | -0.16 ***  (0.04)  [0.000] | -0.16 ***  (0.04)  [0.000] | -0.12 **  (0.04)  [0.001] |
| Perceived COVID-19 risk | | 0.01  (0.02)  [0.648] | 0.01  (0.02)  [0.557] | 0.01  (0.02)  [0.895] |
| **Political views**  **(ref. conservative)** | **Liberal** | -0.26 ***  (0.04)  [0.000] | -0.26 ***  (0.04)  [0.000] | -0.22 ***  (0.04)  [0.000] |
|  | **Neutral** | -0.11 **  (0.04)  [0.002] | -0.11 **  (0.04)  [0.002] | -0.09 **  (0.04)  [0.009] |
| **Social norms** | **Perceived social tightness** | - | -0.01  (0.02)  [0.445] | -0.06 ***  (0.02)  [0.000] |
|  | **Vertical individualism** | - | - | 0.11 ***  (0.02)  [0.000] |
|  | **Horizontal individualism** | - | - | 0.09 ***  (0.02)  [0.000] |
| Other country-level control variables | Country-level case fatality rate | 0.11  (0.03)  [0.238] | 0.11  (0.03)  [0.218] | 0.10  (0.03)  [0.105] |
|  | Country-level per capita income | -0.14  (0.14)  [0.385] | -0.14  (0.14)  [0.381] | -0.23  (0.15)  [0.226] |
| Intercept |  | 0.08  (0.05)  [0.134] | 0.08  (0.05)  [0.118] | -0.01  (0.05)  [0.969] |
| Observations |  | 4260 | 4260 | 4260 |
| AIC |  | 11841.81 | 11849.69 | 11785.40 |
| Pseudo-R² (fixed effects) | | 0.04 | 0.04 | 0.07 |
| Pseudo-R² (total) |  | 0.12 | 0.12 | 0.16 |
| Notes: Standard errors are in round brackets and *p*-values are in square brackets; *** denotes p < 0.001. ** denotes p < 0.01. * denotes p < 0.05. | | | | |

Table S5. Multilevel linear regression models examining the relationships between people’s perceived social benefits and their political views, perceived social tightness, vertical and horizontal individualism, and privacy concerns (n=4260).

| Perceived social benefits | | | | |  |
| --- | --- | --- | --- | --- | --- |
|  |  | Model 3a | Model 3b | Model 3c | Model 3d |
|  |  | Coef.  (S.E.)  [*p*-value] | Coef.  (S.E.)  [*p*-value] | Coef.  (S.E.)  [*p*-value] | Coef.  (S.E.)  [*p*-value] |
| Age group  (ref. 25 – 44 years old) | 18 - 24 years old | -0.02  (0.06)  [0.768] | -0.04  (0.05)  [0.441] | -0.06  (0.05)  [0.253] | -0.08  (0.05)  [0.102] |
|  | 45+ years old | 0.19 ***  (0.03)  [0.000] | 0.18 ***  (0.03)  [0.000] | 0.20 ***  (0.03)  [0.000] | 0.09 ***  (0.03)  [0.000] |
| Gender (ref. male) | Female | -0.03  (0.03)  [0.272] | -0.03  (0.03)  [0.349] | -0.02  (0.03)  [0.549] | 0.02  (0.03)  [0.436] |
| Educational status  (ref. without high educational degree) | High education | 0.06  (0.03)  [0.068] | 0.04  (0.03)  [0.210] | 0.03  (0.03)  [0.295] | 0.06 *  (0.03)  [0.020] |
| COVID-19 infection  (ref. never been infected) | Infected with COVID-19  (self) | 0.02  (0.03)  [0.473] | 0.04  (0.03)  [0.202] | 0.03  (0.03)  [0.297] | 0.07 *  (0.03)  [0.014] |
|  | Infected with COVID-19  (family members) | -0.07 *  (0.04)  [0.048] | -0.05  (0.04)  [0.141] | -0.05  (0.04)  [0.205] | -0.10 **  (0.03)  [0.002] |
| Perceived COVID-19 risk | | 0.19 ***  (0.01)  [0.000] | 0.15 ***  (0.02)  [0.000] | 0.15 ***  (0.02)  [0.000] | 0.15 ***  (0.01)  [0.000] |
| **Political views**  **(ref. conservative)** | **Liberal** | 0.03  (0.04)  [0.429] | 0.09 *  (0.04)  [0.013] | 0.11 **  (0.04)  [0.005] | 0.01  (0.03)  [0.696] |
|  | **Neutral** | -0.16 ***  (0.04)  [0.000] | -0.11 **  (0.03)  [0.002] | -0.09 **  (0.03)  [0.009] | -0.13 ***  (0.03)  [0.000] |
| **Social norms** | **Perceived social tightness** | - | 0.22 ***  (0.02)  [0.000] | 0.22 ***  (0.02)  [0.000] | 0.20 ***  (0.01)  [0.000] |
|  | **Vertical individualism** | - | - | 0.06 ***  (0.02)  [0.000] | 0.11 ***  (0.02)  [0.000] |
|  | **Horizontal individualism** | - | - | -0.08 ***  (0.02)  [0.000] | -0.04 **  (0.01)  [0.002] |
|  | **Privacy concerns** | - | - | - | -0.42 ***  (0.01)  [0.000] |
| Other country-level control variables | Country-level case fatality rate | -0.01  (0.10)  [0.955] | -0.07  (0.25)  [0.863] | -0.01  (0.09)  [0.934] | -0.01  (0.16)  [0.992] |
|  | Country-level per capita income | 0.31  (0.26)  [0.332] | 0.26  (0.29)  [0.533] | 0.29  (0.19)  [0.259] | 0.17  (0.20)  [0.559] |
| Intercept |  | 0.12  (0.10)  [0.444] | 0.17  (0.14)  [0.326] | 0.05  (0.09)  [0.695] | 0.14  (0.10)  [0.258] |
| Observations |  | 4260 | 4260 | 4260 | 4260 |
| AIC |  | 11668.71 | 11469.55 | 11447.02 | 10537.40 |
| Pseudo-R² (fixed effects) | | 0.10 | 0.11 | 0.14 | 0.26 |
| Pseudo-R² (total) | | 0.35 | 0.32 | 0.30 | 0.38 |
| Notes: Standard errors are in round brackets and *p*-values are in square brackets; *** denotes p < 0.001. ** denotes p < 0.01. * denotes p < 0.05. | | | | | |

Table S6. Multilevel linear regression models examining the relationships between people’s acceptance and their political views, perceived social tightness, vertical and horizontal individualism, privacy concerns, and perceived social benefits (n=4260).

| Acceptance | | | | | |
| --- | --- | --- | --- | --- | --- |
|  |  | Model 4a | Model 4b | Model 4c | Model 4d |
|  |  | Coef.  (S.E.)  [*p*-value] | Coef.  (S.E.)  [*p*-value] | Coef.  (S.E.)  [*p*-value] | Coef.  (S.E.)  [*p*-value] |
| Age group  (ref. 25 – 44 years old) | 18 - 24 years old | -0.02  (0.06)  [0.774] | -0.04  (0.05)  [0.438] | -0.06  (0.05)  [0.240] | -0.03  (0.03)  [0.348] |
|  | 45+ years old | 0.15 ***  (0.03)  [0.000] | 0.14 ***  (0.03)  [0.000] | 0.16 ***  (0.03)  [0.000] | -0.03 *  (0.02)  [0.039] |
| Gender (ref. male) | Female | -0.03  (0.03)  [0.341] | -0.02  (0.03)  [0.430] | -0.01  (0.03)  [0.784] | 0.02  (0.02)  [0.136] |
| Educational status  (ref. without a high educational degree) | High education | 0.02  (0.03)  [0.481] | 0.01  (0.03)  [0.918] | 0.01  (0.03)  [0.886] | -0.01  (0.02)  [0.457] |
| COVID-19 infection  (ref. never been infected) | Infected with COVID-19  (self) | -0.02  (0.03)  [0.592] | 0.01  (0.03)  [0.989] | -0.01  (0.03)  [0.782] | -0.02  (0.02)  [0.375] |
|  | Infected with COVID-19  (family members) | -0.13 ***  (0.04)  [0.000] | -0.11 **  (0.04)  [0.003] | -0.10 **  (0.04)  [0.005] | -0.09 ***  (0.02)  [0.000] |
| Perceived COVID-19 risk | | 0.20 ***  (0.01)  [0.000] | 0.16 ***  (0.01)  [0.000] | 0.16 ***  (0.01)  [0.000] | 0.04 ***  (0.01)  [0.000] |
| **Political views**  **(ref. conservative)** | **Liberal** | -0.02  (0.04)  [0.522] | 0.04  (0.04)  [0.292] | 0.06  (0.04)  [0.134] | -0.06 **  (0.02)  [0.001] |
|  | **Neutral** | -0.19 ***  (0.04)  [0.000] | -0.14 ***  (0.03)  [0.000] | -0.12 ***  (0.03)  [0.000] | -0.07 ***  (0.02)  [0.000] |
| **Social norms** | **Perceived social tightness** | - | 0.22 ***  (0.01)  [0.000] | 0.22 ***  (0.02)  [0.000] | 0.05 ***  (0.01)  [0.000] |
|  | **Vertical individualism** | - | - | 0.08 ***  (0.02)  [0.000] | 0.06 ***  (0.01)  [0.000] |
|  | **Horizontal individualism** | - | - | -0.08 ***  (0.02)  [0.000] | -0.01  (0.01)  [0.606] |
|  | **Privacy concerns** | - | - | - | -0.20 ***  (0.01)  [0.000] |
|  | **Perceived social benefits** | - | - | - | 0.73 ***  (0.01)  [0.000] |
| Other country-level control variables | Country-level case fatality rate | -0.14  (0.23)  [0.631] | -0.13  (0.18)  [0.662] | -0.12  (0.20)  [0.730] | -0.08  (0.04)  [0.708] |
|  | Country-level per capita income | 0.04  (0.23)  [0.872] | 0.07  (0.20)  [0.733] | 0.27  (0.26)  [0.436] | 0.04  (0.04)  [0.767] |
| Intercept |  | 0.12  (0.15)  [0.496] | 0.08  (0.14)  [0.587] | 0.25  (0.12)  [0.154] | 0.15 ***  (0.03)  [0.000] |
| Observations |  | 4260 | 4260 | 4260 | 4260 |
| AIC |  | 11615.82 | 11404.56 | 11377.65 | 5938.35 |
| Pseudo-R² (fixed effects) | | 0.07 | 0.11 | 0.12 | 0.77 |
| Pseudo-R² (total) | | 0.20 | 0.21 | 0.30 | 0.77 |
| Notes: Standard errors are in round brackets and *p*-values are in square brackets; *** denotes p < 0.001. ** denotes p < 0.01. * denotes p < 0.05. | | | | | |

Table S7. Multilevel structural equation model examining hypothesis: (1) people’s political views, perceived social tightness, and individualism have direct effects on their privacy concerns, perceived social benefits, and acceptance of the COVID-19 control measures (n=4260).

| Model 5a | | | | |
| --- | --- | --- | --- | --- |
|  |  | **Privacy concern** | **Perceived social benefits** | **Acceptance** |
|  |  | Coef.  (S.E.)  [*p*-value] | Coef.  (S.E.)  [*p*-value] | Coef.  (S.E.)  [*p*-value] |
| Age group  (ref. 25 – 44 years old) | 18 - 24 years old | -0.01 (0.01)  [0.362] | -0.02 (0.01)  [0.224] | -0.02 (0.01)  [0.251] |
|  | 45+ years old | -0.13 * (0.05)  [0.011] | 0.10 * (0.05)  [0.038] | 0.08  (0.05)  [0.098] |
| Gender (ref. male) | Female | 0.04 (0.03)  [0.199] | -0.01 (0.03)  [0.730] | -0.01 (0.03)  [0.905] |
| Educational status  (ref. without a high educational degree) | High education | 0.04 ** (0.01)  [0.001] | 0.02 (0.01)  [0.093] | -0.01 (0.01)  [0.763] |
| COVID-19 infection  (ref. never been infected) | Infected with COVID-19  (self) | 0.05 *** (0.01)  [0.000] | 0.02 (0.02)  [0.385] | -0.01 (0.02)  [0.735] |
|  | Infected with COVID-19  (family members) | -0.06 ** (0.02)  [0.005] | -0.02 (0.02)  [0.163] | -0.05 * (0.02)  [0.023] |
| Perceived pandemic risk | | 0.01  (0.05)  [0.928] | 0.15 **  (0.05)  [0.006] | 0.16 ** (0.05)  [0.002] |
| **Political views**  **(ref. conservative)** | **Liberal** | -0.10 * (0.05)  [0.027] | 0.05 (0.04)  [0.222] | 0.03  (0.04)  [0.497] |
|  | **Neutral** | -0.05 (0.03)  [0.079] | -0.05  (0.03)  [0.061] | -0.06 * (0.03)  [0.033] |
| **Social norms** | **Perceived social tightness** | -0.06 ** (0.03)  [0.009] | 0.23 *** (0.02)  [0.000] | 0.23 *** (0.02)  [0.000] |
|  | **Vertical individualism** | 0.11 *** (0.02)  [0.000] | 0.06 * (0.03)  [0.048] | 0.08 ** (0.03)  [0.002] |
|  | **Horizontal individualism** | 0.09 *** (0.01)  [0.000] | -0.08 * (0.04)  [0.028] | -0.08 * (0.04)  [0.017] |
| Other country-level control variables | Country-level case fatality rate | 0.58 (0.48)  [0.201] | -0.31 (0.35)  [0.388] | -0.61 * (0.24)  [0.011] |
|  | Country-level per capita income | -0.12 (0.54)  [0.817] | 0.02 (0.41)  [0.958] | 0.15 (0.34)  [0.669] |
| Notes: Standard errors are in round brackets and p-values are in square brackets; *** denotes p < 0.001. ** denotes p < 0.01. * denotes p < 0.05.  CFI=0.99, TLI=0.99, RMSEA=0.001, SRMR _individual_=0.002, SRMR _country-level_ = 0.004. | | | | |

Table S8. Multilevel structure equation model examining hypotheses: (1) people’s political views, perceived social tightness, and individualism have direct effects on their privacy concerns, perceived social benefits, and acceptance of the COVID-19 control measures; (2) people’s privacy concerns and perceived social benefits play mediating roles in the effects of people’s political views, individualism, and perceived social tightness on their acceptance of COVID-19 control measures (n=4260).

| Model 5b | | | | |
| --- | --- | --- | --- | --- |
|  |  | **Privacy concern** | **Perceived social benefits** | **Acceptance** |
|  |  | Coef.  (S.E.)  [*p*-value] | Coef.  (S.E.)  [*p*-value] | Coef.  (S.E.)  [*p*-value] |
| Age group  (ref. 25 – 44 years old) | 18 - 24 years old | -0.01 (0.01)  [0.344] | -0.02 (0.01)  [0.231] | -0.01 (0.01)  [0.440] |
|  | 45+ years old | -0.13 * (0.05)  [0.012] | 0.10 * (0.05)  [0.036] | -0.02  (0.02)  [0.287] |
| Gender (ref. male) | Female | 0.04 (0.03)  [0.197] | -0.01 (0.03)  [0.716] | 0.01 (0.01)  [0.216] |
| Educational status  (ref. without a high educational degree) | High education | 0.04 ** (0.01)  [0.001] | 0.02 (0.01)  [0.110] | 0.01 (0.01)  [0.321] |
| COVID-19 infection  (ref. never been infected) | Infected with COVID-19  (self) | 0.05 *** (0.01)  [0.000] | 0.02 (0.02)  [0.396] | -0.01 (0.01)  [0.255] |
|  | Infected with COVID-19  (family members) | -0.05 ** (0.02)  [0.008] | -0.02 (0.02)  [0.169] | -0.05 *** (0.01)  [0.000] |
| Perceived COVID-19 risk | | -0.01  (0.04)  [0.901] | 0.15 **  (0.02)  [0.008] | 0.05 *** (0.01)  [0.000] |
| **Political views**  **(ref. conservative)** | **Liberal** | -0.10 * (0.05)  [0.026] | 0.05 (0.04)  [0.233] | -0.03* (0.01)  [0.013] |
|  | **Neutral** | -0.05  (0.03)  [0.073] | -0.05 * (0.03)  [0.045] | -0.04 ** (0.01)  [0.001] |
| **Social norms** | **Perceived social tightness** | -0.06 ** (0.03)  [0.009] | 0.23 *** (0.02)  [0.000] | 0.06 *** (0.01)  [0.000] |
|  | **Vertical individualism** | 0.10 *** (0.02)  [0.000] | 0.06 (0.03)  [0.087] | 0.06 *** (0.01)  [0.000] |
|  | **Horizontal individualism** | 0.09 *** (0.01)  [0.000] | -0.08 * (0.04)  [0.026] | -0.01 (0.01)  [0.716] |
|  | **Privacy concerns** | - | - | -0.22 *** (0.03)  [0.000] |
|  | **Perceived social benefits** | - | - | 0.78 *** (0.02)  [0.000] |
| Other country-level control variables | Country-level case fatality rate | 0.57 (0.47)  [0.201] | -0.31 (0.36)  [0.399] | -0.61 * (0.24)  [0.012] |
|  | Country-level per capita income | -0.14 (0.55)  [0.817] | 0.02 (0.41)  [0.958] | 0.15 (0.34)  [0.669] |
| Notes: Standard errors are in round brackets and p-values are in square brackets; *** denotes p < 0.001. ** denotes p < 0.01. * denotes p < 0.05.  CFI=0.99, TLI=0.99, RMSEA=0.001, SRMR _individual_=0.002, SRMR _country-level_ = 0.004. | | | | |

Table S9. Multilevel structure equation model examining hypotheses: (1) people’s political views, perceived social tightness, and individualism have direct effects on their privacy concerns, perceived social benefits, and acceptance of the COVID-19 control measures; (2) people’s privacy concerns and perceived social benefits play mediating roles in the effects of people’s political views, individualism, and perceived social tightness on their acceptance of COVID-19 control measures; (3) people’s perceived social benefits play a mediating role in the effects of people’s privacy concerns on their acceptance of COVID-19 control measures (n=4260).

| Model 5c | | | | |
| --- | --- | --- | --- | --- |
|  |  | **Privacy concern** | **Perceived social benefits** | **Acceptance** |
|  |  | Coef.  (S.E.)  [*p*-value] | Coef.  (S.E.)  [*p*-value] | Coef.  (S.E.)  [*p*-value] |
| Age group  (ref. 25 – 44 years old) | 18 - 24 years old | -0.01 (0.01)  [0.362] | -0.02 (0.01)  [0.148] | -0.01 (0.01)  [0.450] |
|  | 45+ years old | -0.13 ** (0.05)  [0.009] | 0.05  (0.03)  [0.118] | -0.02 (0.02)  [0.296] |
| Gender (ref. male) | Female | 0.04 (0.03)  [0.199] | 0.01 (0.02)  [0.667] | 0.01 (0.01)  [0.199] |
| Educational status  (ref. without a high educational degree) | High education | 0.04 ** (0.01)  [0.001] | 0.03 *** (0.01)  [0.000] | -0.01 (0.01)  [0.307] |
| COVID-19 infection  (ref. never been infected) | Infected with COVID-19  (self) | 0.05 *** (0.01)  [0.000] | 0.04 (0.02)  [0.071] | -0.01 (0.01)  [0.260] |
|  | Infected with COVID-19  (family members) | -0.05 ** (0.02)  [0.005] | -0.04 *** (0.01)  [0.000] | -0.04 *** (0.01)  [0.000] |
| Perceived pandemic risk | | -0.01  (0.04)  [0.928] | 0.15 ***  (0.03)  [0.000] | 0.05 *** (0.01)  [0.000] |
| **Political views**  **(ref. conservative)** | **Liberal** | -0.10 * (0.05)  [0.027] | 0.01 (0.02)  [0.862] | -0.03 ** (0.01)  [0.009] |
|  | **Neutral** | -0.05  (0.03)  [0.079] | -0.07 * (0.03)  [0.028] | -0.04 ** (0.01)  [0.001] |
| **Social norms** | **Perceived social tightness** | -0.06 ** (0.03)  [0.009] | 0.20 *** (0.02)  [0.000] | 0.05 *** (0.01)  [0.000] |
|  | **Vertical individualism** | 0.10 *** (0.02)  [0.000] | 0.10 *** (0.03)  [0.000] | 0.06 *** (0.01)  [0.000] |
|  | **Horizontal individualism** | 0.09 *** (0.01)  [0.000] | -0.05 (0.04)  [0.208] | -0.01  (0.01)  [0.726] |
|  | **Privacy concerns** | - | -0.42 ***  (0.03)  [0.000] | -0.20 *** (0.02)  [0.000] |
|  | **Perceived social benefits** | - | - | 0.73 *** (0.02)  [0.000] |
| Other country-level control variables | Country-level case fatality rate | 0.58 (0.47)  [0.201] | -0.31 (0.35)  [0.388] | -0.61 * (0.24)  [0.011] |
|  | Country-level per capita income | -0.14 (0.55)  [0.817] | 0.02 (0.41)  [0.958] | 0.15 (0.34)  [0.668] |
| Notes: Standard errors are in round brackets and p-values are in square brackets; *** denotes p < 0.001. ** denotes p < 0.01. * denotes p < 0.05.  CFI=1.00, TLI=1.00, RMSEA=0.000, SRMR _individual_=0.002, SRMR _country-level_ = 0.004. | | | | |


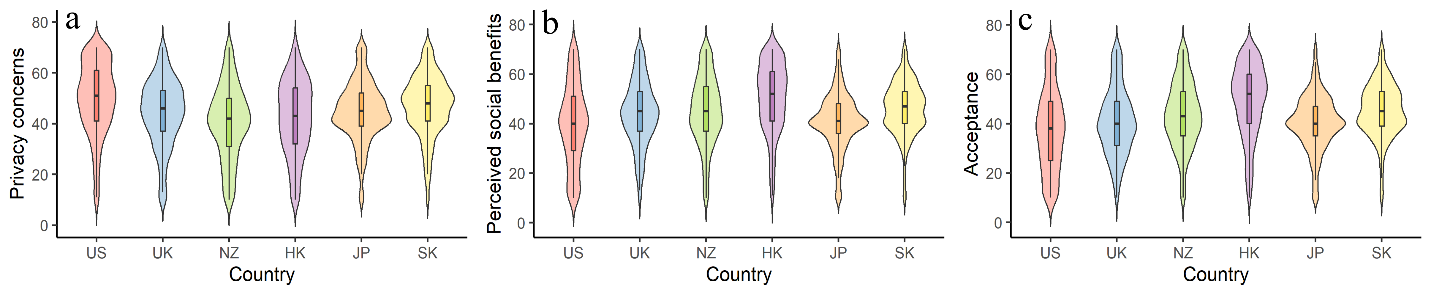


Figure S1. Statistical distributions of people’s views on the COVID-19 control measures in the six study areas. (a) Privacy concerns; (b) Perceived social benefits (S-B); and (c) Acceptance.


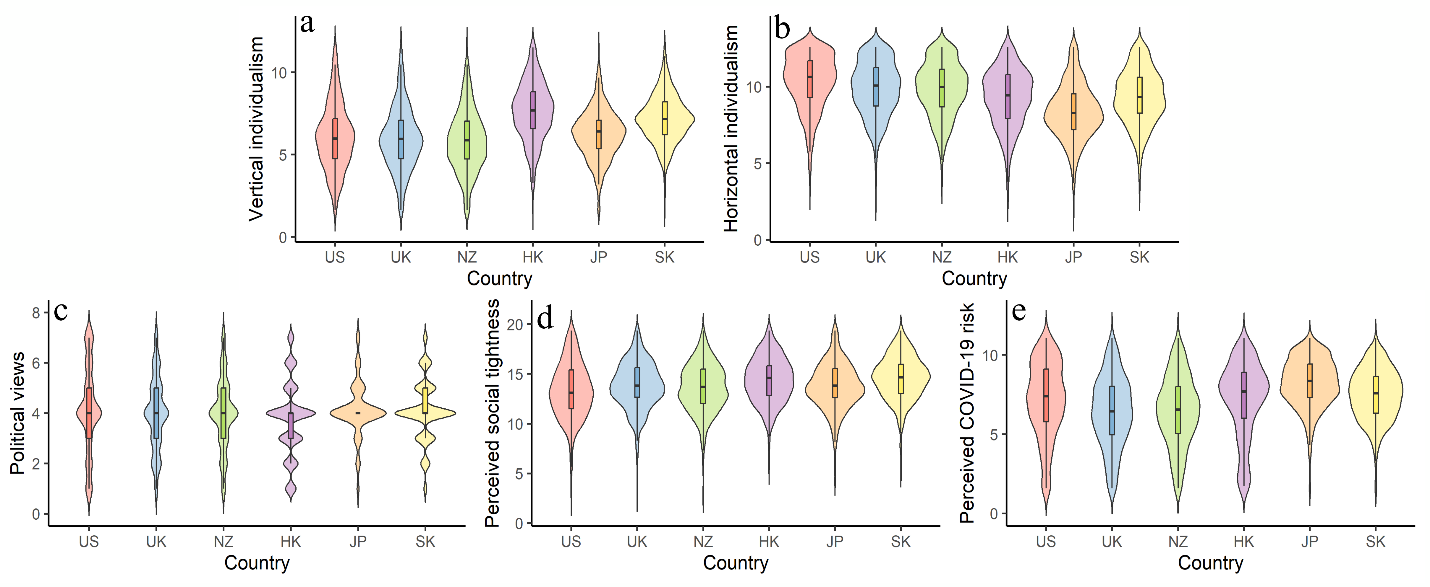


Figure S2. Statistical distributions of people’s vertical and horizontal individualism, political views, perceived social tightness, and perceived COVID-19 risk across the six study areas. (a) vertical individualism; (b) horizontal individualism; (c) political views; (d) perceived social tightness; (e) perceived COVID-19 risk.


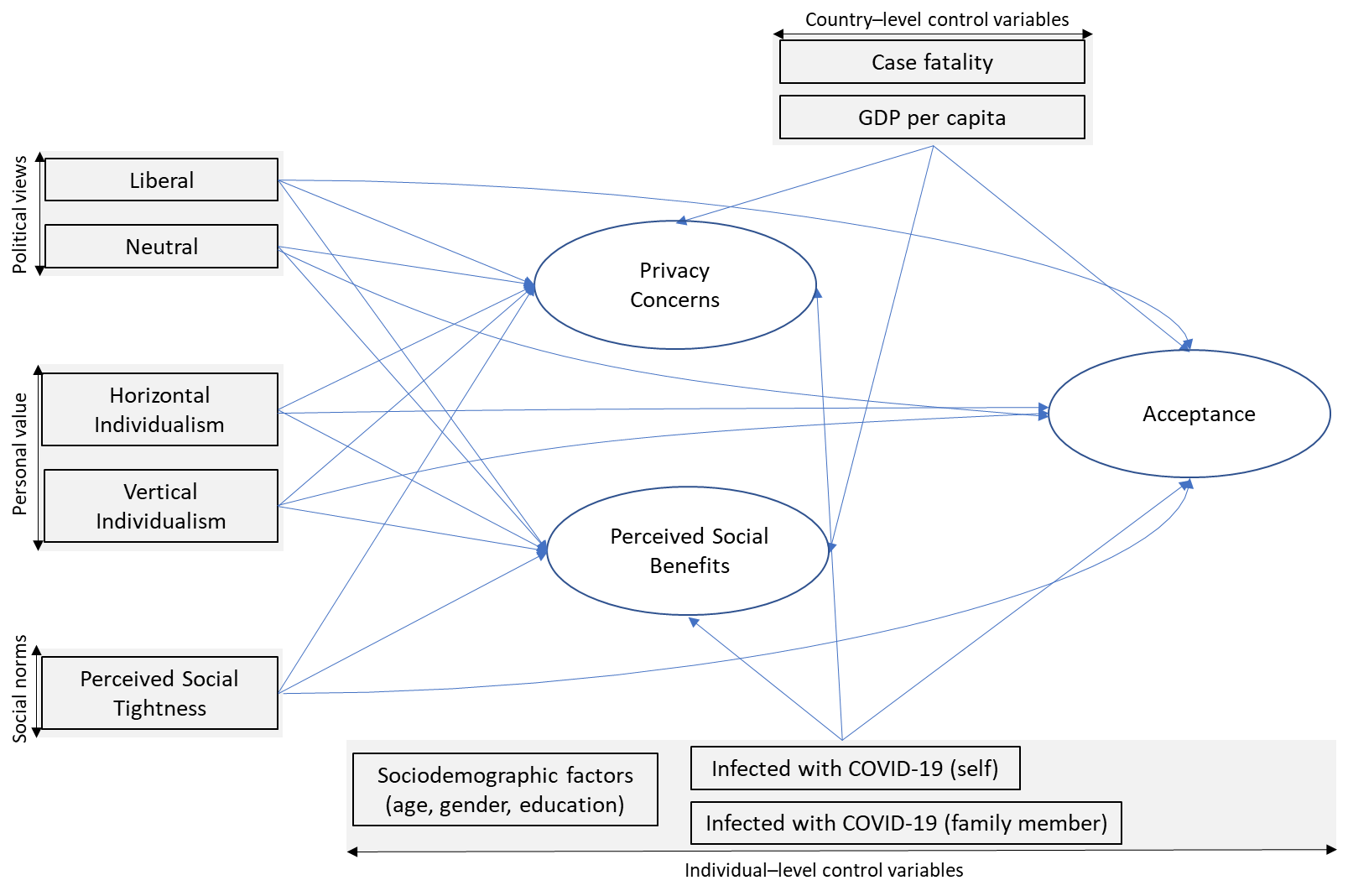


Figure S3. Conceptual model (Model 5a) for examining the hypothesis: (1) people’s political views, perceived social tightness, and individualism have direct effects on their privacy concerns, perceived social benefits, and acceptance of the COVID-19 control measures.


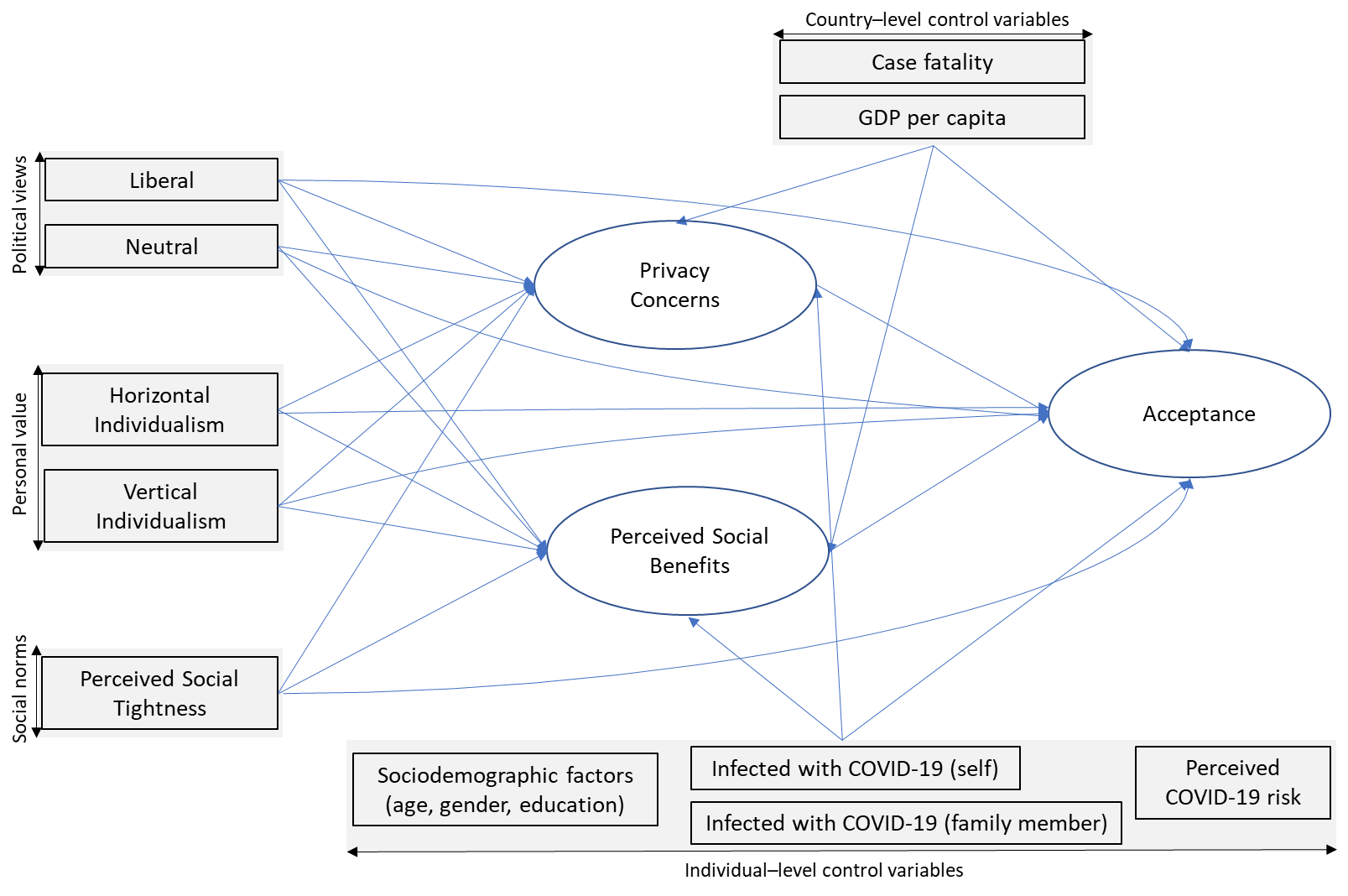


Figure S4. Conceptual model (Model 5b) for examining the hypotheses: (1) people’s political views, perceived social tightness, and individualism have direct effects on their privacy concerns, perceived social benefits, and acceptance of the COVID-19 control measures; (2) people’s privacy concerns and perceived social benefits play mediating roles in the effects of people’s political views, individualism, and perceived social tightness on their acceptance of COVID-19 control measures.


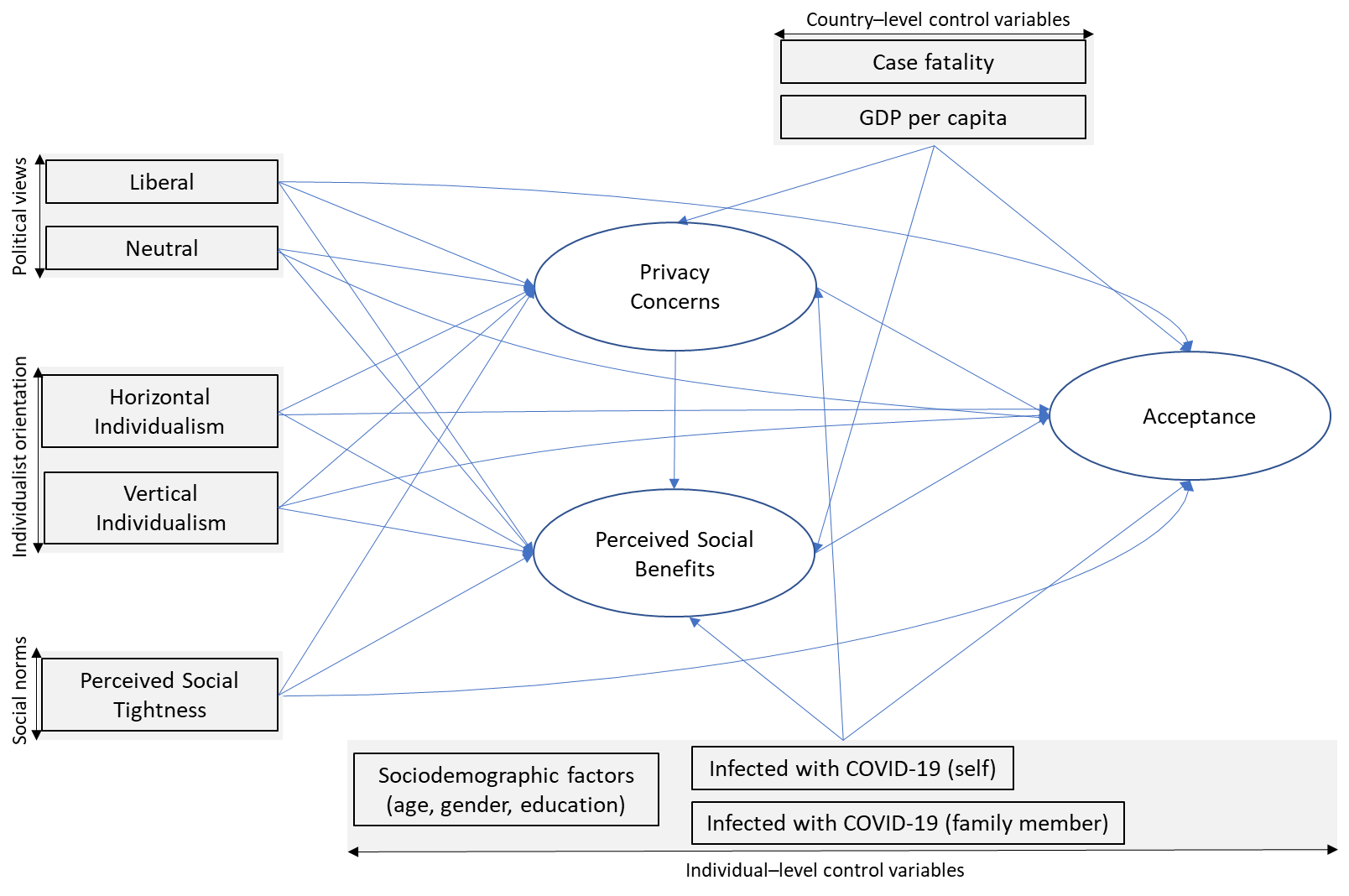


Figure S5. Conceptual model (Model 5c) for examining the hypotheses: (1) people’s political views, perceived social tightness, and individualism have direct effects on their privacy concerns, perceived social benefits, and acceptance of the COVID-19 control measures; (2) people’s privacy concerns and perceived social benefits play mediating roles in the effects of people’s political views, individualism, and perceived social tightness on their acceptance of COVID-19 control measures; (3) people’s perceived social benefits play a mediating role in the effects of people’s privacy concerns on their acceptance of COVID-19 control measures.
